# ISG15 mediates the function of extracellular vesicles in promoting ovarian cancer progression and metastasis

**DOI:** 10.1002/jex2.92

**Published:** 2024-01-31

**Authors:** Kalpana Deepa Priya Dorayappan, Vincent Wagner, Dongju Park, Meghan M. Newcomer, Michelle D. S. Lightfoot, Deepika Kalaiyarasan, Takahiko Sakaue, Wafa Khadraoui, Lianbo Yu, Qi‐En Wang, G. Larry Maxwell, David O'Malley, Raphael E. Pollock, David E. Cohn, Karuppaiyah Selvendiran

**Affiliations:** ^1^ Division of Gynecologic Oncology, Department of Obstetrics and Gynecology Comprehensive Cancer Center, The Ohio State University Wexner Medical Center Columbus Ohio USA; ^2^ Molecular Genetics, Comprehensive Cancer Center The Ohio State University Wexner Medical Center Columbus Ohio USA; ^3^ Department of Anatomy, School of Medicine Case Western Reserve University Cleveland Ohio USA; ^4^ Division of Gynecologic Oncology, Department of Obstetrics and Gynecology NYU Langone Health/Perlmutter Cancer Center New York New York USA; ^5^ Division of Gastroenterology, Department of Medicine Kurume University School of Medicine Kurume Japan; ^6^ Department of Biomedical Informatics The Ohio State University Wexner Medical Center Columbus Ohio USA; ^7^ Department of Radiation Oncology The Ohio State University Columbus Ohio USA; ^8^ Inova Women's Service Line and the Inova Schar Cancer Institute Falls Church Virginia USA; ^9^ Division of Surgical Oncology, The James Comprehensive Cancer Center Ohio State University Columbus Ohio USA

**Keywords:** ascites, extracellular vesicles, ISG15, ISGylation, metastasis and biomarker, ovarian cancer

## Abstract

The interferon stimulated gene 15 (ISG15), a ubiquitin like protein and its conjugates have been implicated in various human malignancies. However, its role in ovarian cancer progression and metastasis is largely unknown. In high grade serous ovarian cancer (HGSOC), ascites is the major contributor to peritoneal metastasis. In this study, we identified significantly elevated ISG15 protein expression in HGSOC patient ascites, ascites derived primary ovarian cancer cells (POCCs), POCC small extracellular vesicles (sEVs) as well as metastatic tissue. Our results demonstrates that ISG15 increases exocytosis in ascites‐derived POCCs by decreasing the endosome‐lysosomal fusion, indicating a key role in sEV secretion. Further, knockdown (KD) of ISG15 resulted in a significant decrease in vesicles secretion from HGSOC cells and *in* *vivo* mouse models, leading to reduced HGSOC cell migration and invasion. Furthermore, our pre‐clinical mouse model studies revealed the influence of vesicular ISG15 on disease progression and metastasis. In addition, knockdown of ISG15 or using the ISG15 inhibitor, DAP5, in combination therapy with carboplatin showed to improve the platinum sensitivity in‐vitro and reduce tumour burden in‐vivo. We also found that ISG15 expression within sEV represents a promising prognostic marker for HGSOC patients. Our findings suggest that ISG15 is a potential therapeutic target for inhibiting progression and metastasis in HGSOC and that vesicular ISG15 expression could be a promising biomarker in the clinical management of ovarian cancer.

**Significance**: High‐grade serous ovarian cancer (HGSOC) has high morbidity and mortality rates, but its progression and metastasis are still poorly understood, and there is an urgent need for early detection and targeted therapies. Our study presents novel findings that implicate ISG15‐mediated vesicular proteins in the advancement and spread of HGSOC. These results offer pre‐clinical evidence of potential new molecular targets, prognostic markers and therapeutic strategies for HGSOC that could ultimately enhance patient survival.

## 1 INTRODUCTION

Ovarian cancer (OC) remains the primary cause of gynaecologic cancer‐related deaths in the United States, with high grade serous ovarian cancer (HGSOC) being the most frequently encountered histologic subtype. It is anticipated that by 2023, there will be over 221,500 new cases, leading to approximately 134,770 deaths (Siegel et al., 2022, 2023). The major factors contributing to poor survival are early peritoneal spread/metastasis, eventual recurrence, and ultimately chemoresistance which accounts for 80%–90% of all OC deaths (Davis et al., 2014; Holmes, 2015; Lengyel, 2010). Clinical evidence shows that malignant ascites is routinely observed in OC and that ascites promotes disease progression to an advanced stage (Ahmed & Stenvers, 2013; Saini et al., 2017; Smolle et al., 2014). Ascites is recognized as a major contributor of peritoneal metastasis, modulation of the tumour microenvironment, remodelling of the extracellular matrix, immune evasion and chemoresistance. There is emerging evidence that a variety of growth factors, cytokines and small extracellular vesicles (sEVs) are present in the ascites of ovarian cancer patients that contribute to cell proliferation, angiogenesis, as well as immune and inflammatory responses (Dorayappan et al., 2019; Dorayappan et al., 2016; Dorayappan et al., 2018; Vergauwen et al., 2021). Therefore, understanding the underlying biologic mechanisms of peritoneal dissemination is essential for regulation of this distinct form of tumour metastasis and may be key to better therapies.

Extracellular vesicles (sEVs) are nano‐sized (30–120 nm) vesicles released by a variety of cells and are generated within the endosomal system (Dorayappan et al., 2016; Kalluri, 2016; Ruivo et al., 2017). Cancer sEVs are considered potential mediators of tumour progression and metastasis (Hoshino et al., 2015; Rodrigues et al., 2019). Previous studies show that these sEVs facilitate the peritoneal dissemination of ovarian cancer through the transfer of oncogenic contents such as proteins and nucleic acids.

Interferon‐Stimulated Gene 15 (ISG15) is emerging as an important oncogene and a potential diagnostic and therapeutic target for cancer (Desai, 2015; Kariri et al., 2021; Zhou et al., 2017). ISG15 protein exerts its function in its free form and by covalently linking (conjugation) to target substrates by a process termed ISGylation (Alcala et al., 2020). Ubiquitin‐specific protease 18 (USP18) constitutes the major ISG15 specific protease of the known deubiquitinases which counteracts ISG15 conjugation (Basters et al., 2017). Recent studies have shown ISGylation of cellular proteins alters trafficking and sEV's secretion (Villarroya‐Beltri et al., 2016). ISG15 expression has been reported to be upregulated in several cancer cells, such as melanoma, breast, prostate, hepatocellular, lung and nasopharyngeal cancer (Andersen et al., 2006; Kariri et al., 2021; Li et al., 2014; Sainz et al., 2014). Relative to ubiquitin, the biological function of ISG15 is still poorly understood. A recent study identified that ISGylation and ISG15 conjugation of multivesicular body proteins regulate sEV secretion in cancer cells (Villarroya‐Beltri et al., 2016) .

In the current study we sought to determine the role of ISG15 in regulating ovarian cancer progression and metastasis by studying the effects of ISGylation on EV release, which are critical for the spread of ovarian cancer. The aim of this study is to provide molecular insights related to ISG15‐mediated EV secretion in the tumour microenvironment of malignant ascites to identify novel treatment and monitoring strategies in HGSOC.

## 2 METHODS & MATERIALS

### 2.1 Cell lines

Immortalized ovarian surface epithelial cells (IOSE385 and IOSE386), (Gifted by Dr. Clara Salamanca, BCCRC, Vancouver, BC, Canada), immortalized high grade serous ovarian cancer (HGSOC) human cell lines (OVCAR‐3 and 4) were utilized. Immortalized patient derived ascites cells (TR127 & TR182) (Gifted by Dr. G. Mor of Yale University) derived from recurrent, platinum chemotherapy resistant ovarian cancer were used in the overexpression and knockdown studies. In addition, ascites derived primary ovarian cancer cells (POCCs) isolated from different ovarian cancer patients were cultured as described previously (Dorayappan et al., 2018). All the above‐mentioned cell lines were utilized to demonstrate the varied patterns of tumour progression and metastasis in immortalized versus primary ovarian cancer cells isolated from patient ascites. We confirmed mycoplasma activity using ATCC Universal Mycoplasma Detection Kit in all cell lines every 2 months. Once the frozen cells were thawed, they were passaged for a maximum of five times and discarded thereafter and a fresh vial was thawed.

### 2.2 ISG15‐overexpression (OE) and knockdown (KD) studies

To establish a stable ISG15 ‐overexpressing TR127 cell line, the cells were seeded in the manner of 1.5×10^5^ cells in 6‐well plates at day 1 prior to transfection. Transfection was performed using Human Tagged ORF Clone (Cat#RG201235) containing the ISG15 overexpression protein construct cloned into the pCMV6‐AC‐GFP and control empty vector using turbofectin DNA transfection reagent from ORIGENE following the manufacturer's instructions. Following 48 h of transfection, the cells were treated with 100 μg/mL of G418 (Sigma‐Aldrich) every 2 days for 10 days total to establish a stable cell line. To ensure the stable knockdown of ISG15 in the POCC cells, TR127 cells were transfected with pRS vector containing four unique 29mer shRNA constructs from ORIGENE alongside a negative scrambled shRNA for control according to manufacturer's protocol (OriGene Technologies, Inc.). After transfection, the cells were incubated for 2 days followed by selection with 1–2 μg/mL puromycin for 3–5 days. Thereafter, cells were cultured with normal media. The resultant ISG15 –OE and KD cells were used for various experiments.

### 2.3 Transmission electron microscopy (TEM)

The ovary and metastatic ovarian tumour tissues were processed for TEM imaging as follows: The tissues were dissected and fixed in 2.5% glutaraldehyde in 0.1 M phosphate buffer for at least 24 h at 4°C. Likewise, OSE cells and POCCs were also cultured on permanox chamber slides and processed for TEM. The cells were washed and then fixed with 2.5% glutaraldehyde in 0.1 M phosphate buffer for 30 min at room temperature. Both ovary tissue and cell samples were postfixed with 1% osmium tetroxide and then enbloc stained with 1% aqueous uranyl acetate, dehydrated in a graded series of ethanol, and embedded in Eponate 12 epoxy resin (Ted Pella Inc., Redding, CA). Ultrathin sections were cut with a Leica EM UC6 ultramicrotome (Leica microsystems Inc., Deerfield, IL), collected on copper grids. Images were acquired with an FEI Technai G2 Spirit transmission electron microscope (Thermo Fisher Scientific, Waltham, MA), and a Macrofire (Optronics, Inc., Chelmsford, MA) digital camera and AMT image capture software.

### 2.4 Baculovirus transduction

Culture medium from TR127 cells was aspirated after 24 h, and fresh medium containing Cell Light Late Endosomes‐GFP, BacMam 2.0 (Cat# C10588) was added to reach a final concentration of 30 particles per cell, as suggested by the manufacturer's protocol. The cells were gently shaken at 37°C for 1 h to maximize transduction efficiency, and then cultured for another 16−18 h before confocal analysis for sub‐cellular co‐localization with lysosomes.

### 2.5 ISG15 pull down assay

To trace the ISGylation of STAT3 and TSG101 proteins in the cell lysates, PureProteome Protein G Magnetic Bead System (Cat# LSKMAGAG10; Millipore Sigma) was added to the ISG15 immunoprecipitated cell lysates after 12 h precipitation. The beads were then washed and boiled to release the bound sample precipitates. After washing the beads, the ISGylated proteins were subjected to immunoblot for PSTAT3‐Tyr705, ISG15 and TSG101 with their respective primary antibodies.

### 2.6 Cell surface biotinylation for protein trafficking

To assess protein recycling in cells, we performed a standard cell surface biotinylation protocol (explained in Sup. Methods) in one group of cells, with modifications such as re‐heating to allow recycling of some of the internalized, biotin tagged proteins after stripping the biotin off the un‐endocytosed surface proteins. Finally, by calculating the difference between the internalized proteins before and after recycling, we were able to quantify percent of proteins recycled back to the membrane (See the remaining methods in Supplementary Section).

## 3 RESULTS

### 3.1 Determining ISG15 expression and the ISGylation pattern in ovarian cancer patient samples

**FIGURE 1:**
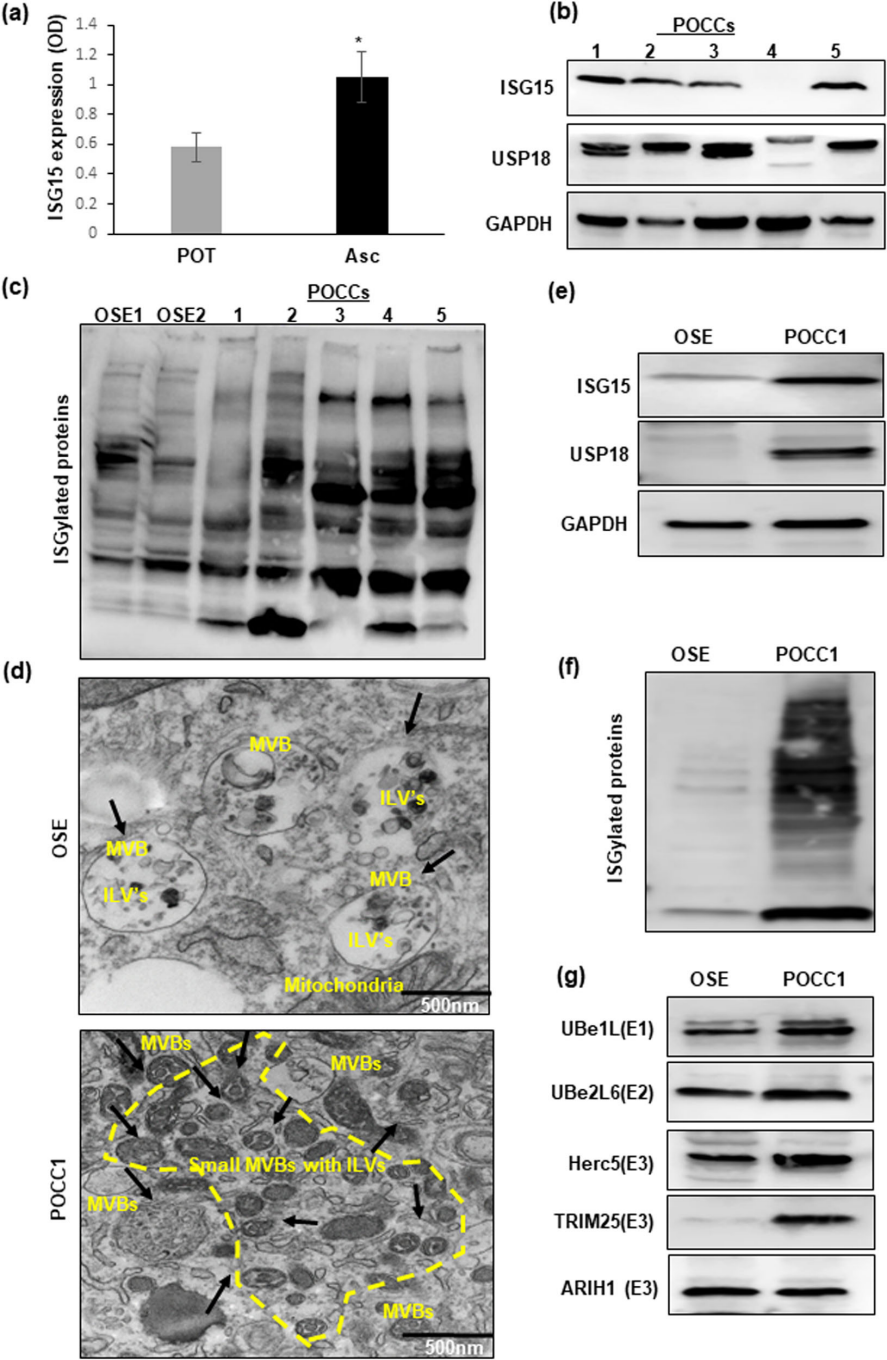
Expression of ISG15 and ISGylation enzymes in patient ascites and ascites derived cells: (a and b) Elevated ISG15 expression shown in HGSOC patient ascites and primary ovarian tumours (POT) by ELISA (*n* = 16) and in different primary ascites derived ovarian cancer cells (POCCs) by western blot (*n* = 5). (c) Comparison of ISGylated proteins in normal ovarian surface epithelial cells (OSE1&2) and patient derived ascites cells (POCC1‐5) (*n* = 5). (d) TEM images of extracellular vesicles in normal OSE cells and in ascites cells (POCC1) (*n* = 3). (e) Western blot comparison of ISG15 and USP18 expression in OSE cells and POCCs (*n* = 3). (f and g) Comparison of ISGylation profile and ISGylation enzymes’ expression in normal OSE cells and POCCs.

To determine whether ISG15 expression and ISGylation play a role in ovarian cancer tumorigenesis, we measured the expression of ISG15 in the primary ovarian tumours and ascites of patients with HGSOC by ELISA. Also, we analysed the ISGylation profiles and ISG15 levels in ascites derived primary ovarian cancer cells (POCCs) by western blot. We found ISG15 expression and ISGylated protein profiles to be highly elevated in both patient ascites and POCCs when compared to normal ovarian surface epithelial cells (OSE) (Figure 1a‐c; Figure S1a). We also found increased ISG15 expression and an increased ISGylation profile in HGSOC patient tissues (Figure S1b) and in tumour and ascites derived from an orthotopic ovarian cancer mouse model (Figure S2a,b) along with USP18 and ISGylating enzyme TRIM25. Further, we have identified densely packed multivesicular bodies in POCCs when compared with OSE cells (Figure 1d; Figure S3a) by TEM analysis. Also, we have confirmed the increased expression of ISG15 and its only known regulator, USP18, in POCCs by western blot (Figure 1e,f) and confocal microscopy (Figure S3b) when compared to OSE cells. In addition, the ISGylated products and the enzymes (E1, E2 and E3 ligases) involved in the ISGylation cycle in the ascites derived POCCs were also increased when compared with OSE cells (Figure 1g). Our results indicate that the free ISG15 and the ISGylation profile along with the ISGylation cycle enzymes are dependent on the cellular context and it is aberrantly expressed and more predominant in HGSOC ascites cells.

### 3.2 Determining the role of ISG15 expression in vesicle secretion and sEV proteins on ovarian cancer cell migration and invasion in‐vitro

**FIGURE 2:**
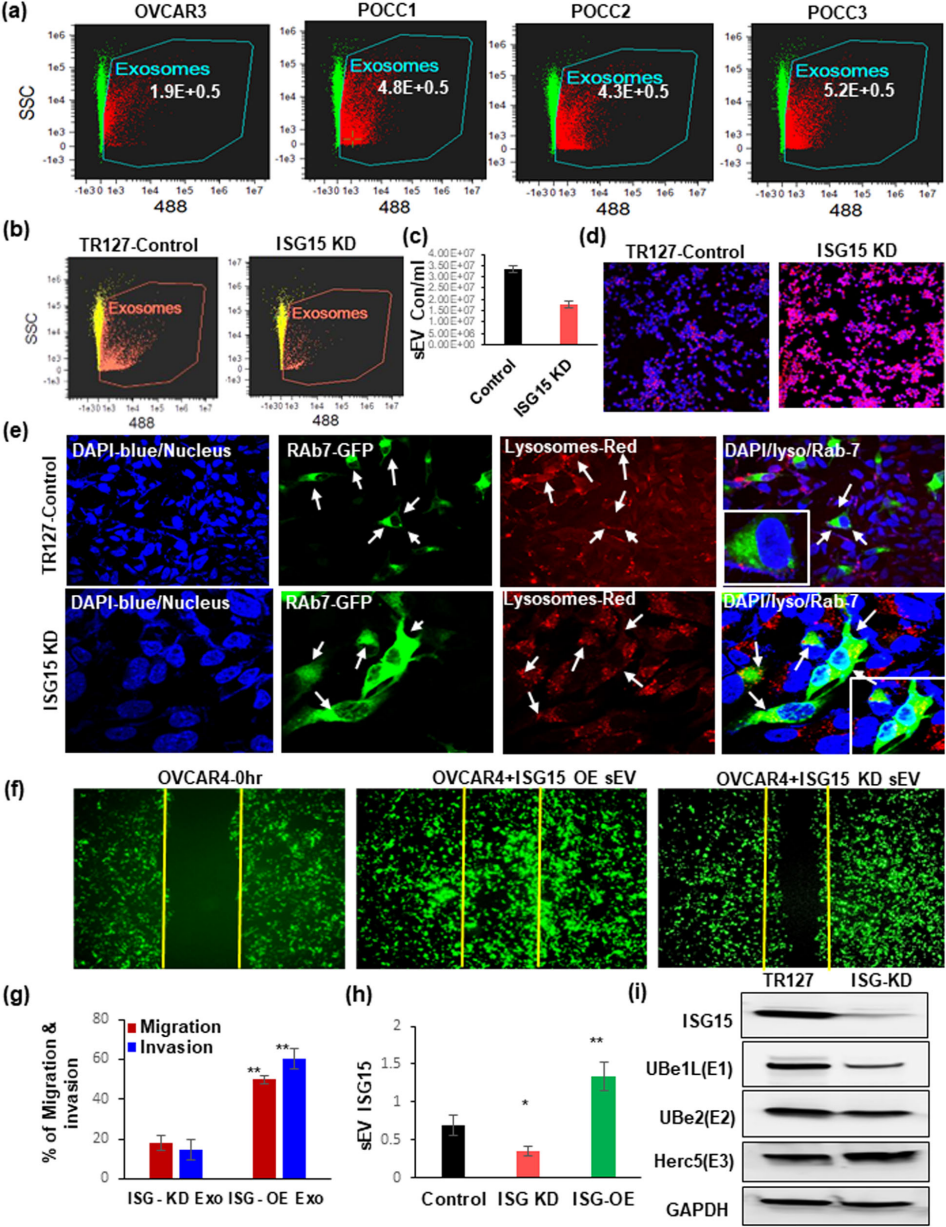
Extracellular vesicles’ concentration and their ISG15 expression influence ovarian cancer migration and invasion: (a) Comparison of small extracellular vesicle (sEV) quantification in immortalized HGSOC cell line –OVCAR3 and ascites derived POCCs by Image stream analysis (ISA) (*n* = 3 samples ± SD). (b and c) Knockdown of ISG15 in TR127‐ at 24 h decreases extracellular vesicle population as analysed by ISA (*n* = 3 samples ± SD). (d) Analysis of the lysosomal staining in ISG15‐KD TR127 cells at 24 h by confocal microscopy. (e) Co‐localization of Rab7‐GFP late endosomes (green) with lysosomes (red) observed in TR127‐ISG‐15‐KD cells after 24 h after transfection when compared with TR127 control cells at 40× magnification with 3× zoom images (inner square) in the merged channels. (f and g) Cell migration in vitro: Comparison of the wound healing effect in GFP labelled OVCAR4 cells after internalization of vesicles isolated from TR127 –ISG15 over expression (OE) or knockdown (KD) cells after 48 h. (h) Expression of vesicular ISG15 in ISG‐OE and ‐KD TR127 cells when compared to control TR127 cells at 24 h. (i) Influence of ISG15 knockdown in TR127 cells on the ISGylation enzymes by western blot analysis.

Previous studies have shown that ISgylation modulates the vesicle trafficking and with our results showing increased vesicles and multivesicular body formations in POCCs than in OSE cells, we sought to quantify the vesicles released from these cells by image stream flowcytometry analysis (ISA). Further, on quantifying the vesicles released, ascites derived POCCs had significantly higher vesicles’ concentration in the conditioned medium than the immortalized HGSOC cells‐OVCAR3 or normal OSE cells (Figure 2a; Figure S4). Further, to implicate the role of ISG15 in vesicle release, we used the immortalized POCC‐TR127‐ISG15‐KD cells and observed a significant decrease in the vesicle release by ISA (Figure 2b,c; Figure S5) along with increased lysosomal expression in ISG15‐KD cells implying that ISG15 could regulate the vesicle release through lysosomal pathway (Figure 2d). Further we also show that ISG15KD in TR127 increases the dynamic cellular process of endo‐lysosomal fusion in POCC cells by transfecting them with the fusion construct of Rab7a and emGFP and co‐localizing it with lysosomes using lyso tracker red by Confocal microscopy (Figure 2e; Figure S6). Future studies are warranted to gain a better understanding of the mechanism behind this event.

To evaluate the role of vesicular ISG15 in cell migration, we first evaluated the uptake of exo‐glow green labelled vesicles isolated from TR127 cells within OVCAR4 cells (Figure S7a). Additionally, we found that the vesicles isolated from TR127 with ISG15 overexpression (ISG‐OE) (Figure S7b) significantly enhanced the migration and invasion potential of GFP‐labelled OVCAR4 than the vesicles isolated from the TR127‐ISG‐KD cells (Figure 2f,g). Furthermore, we confirmed an increased expression of ISG15 within the vesicles released from the TR127‐ISG‐OE cells compared with that from the TR127‐ISG‐KD cells (Figure 2h). When looking specifically at the effects of TR127‐ISG‐KD on ISGylation cycle enzymes, we found that the ISG15 activating enzymes, Ube1L (E1) and Ube2L6 (E2) were decreased, but the substrate‐conjugating E3 enzyme, Herc5, was significantly increased (Figure 2i). These results suggest that ISG15 expression and the ISGylation patterns of proteins in ascites cells modulate vesicle secretion and that vesicular ISG15 plays a key role in cell migration in in‐vitro studies. Also, the substrate‐conjugating enzymes (E3 ligases) may be involved in the conjugation of critical oncogenic substrates with ISG15 preventing their lysosomal clearance and therefore promoting stability to drive tumour progression and metastases in HGSOC.

### 3.3 STAT3 is a target for ISGylation

**FIGURE 3:**
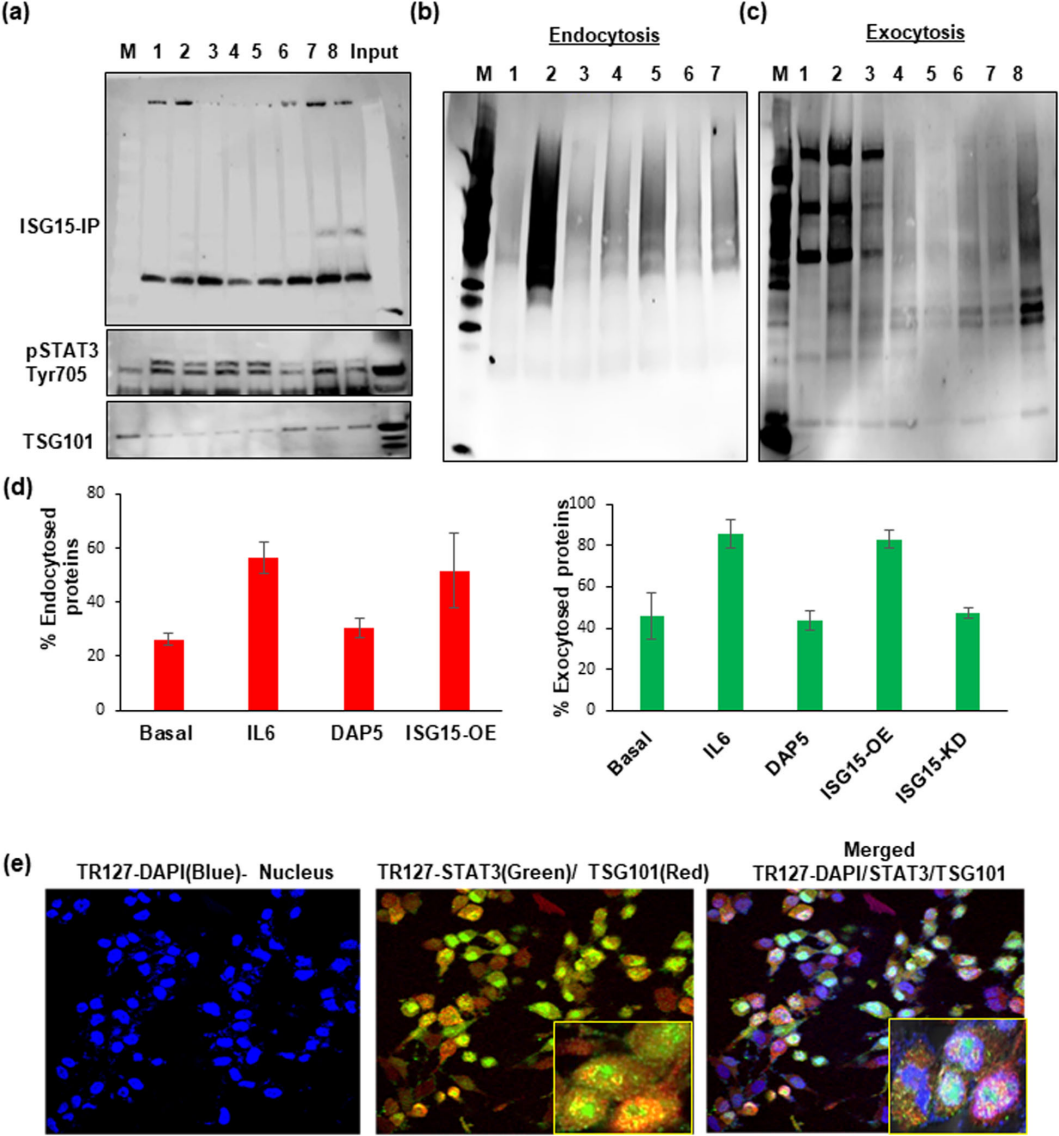
ISGylation of STAT3 and its interaction with ISG15 modulate the vesicle secretion. (a) Pull down of ISG15 by immunoprecipitation in different POCC cells cultured from different patient ascites (Lane 2‐8) along with Input and Marker lane (M) shows the ISGylation of STAT3 and TSG101,a MVB protein, when compared with immortalized HGSOC cancer cells (OVCAR4‐ Lane1) at 24 h after culture. (b) Cell surface biotinylation assay: Endocytosis in TR127 cells at 24 h shows the influence of STAT3 and ISG15 OE and inhibition in protein endocytosis in Basal (Lane 4), IL6 treated cells for STAT3 activation (Lane 5), ISG15 inhibition by DAP5 treatment (Lane 6), and ISG‐OE cells (Lane 7) as compared to their controls‐untreated (Lane 1), biotinylating control (Lane 2), strip control (Lane 3) and marker lane (M). (c) Exocytosis measured in TR127 cells at 24 h in different treatment conditions in Basal (Lane 4), IL6 treatment (Lane 5), ISG15 inhibition by DAP5 treatment (Lane 6), ISG‐OE cells (Lane 7) and ISG15 KD cells (Lane 8) as compared to their controls‐untreated (Lane 1), biotinylating control (Lane 2), strip control (Lane 3) and marker lane (M). (d) Graph showing the % of endo‐ and exocytosed proteins in different treatment conditions described above. (e) Confocal microscopy showing co‐localization of STAT3 and TSG101 in TR127 cells at 24 h cell culture by confocal microscopy at 40× magnification with 3× zoom images (inner square) in the merged channels.

Our previously published (Saini et al., 2017) results showed that the phosphorylated form of STAT3 Tyr705 is highly elevated in ascites and in POCCs when compared to OSE cells (Figure S8a,b). To determine if pSTAT3 was a target for ISGylation that contributes to its increased stability and accumulation in ascites, we evaluated ISG15 pull‐down by immunoprecipitation and confirmed that pSTAT3 was ISGylated in ascites derived POCCs (Figure 3a: Lane‐2–9) when compared to the immortalized, less aggressive HGSOC cells, OVCAR4 (Figure 3a: Lane‐1). Further, we found decreased ISGylation of TSG101, a multivesicular body (MVB) protein, in POCCs (Figure 3a: Lane‐2–9) as compared to OVCAR4 cells (Figure 3a: Lane‐1). TSG101‐positive MVBs are not marked for lysosomal degradation and the cargo within these vesicles may contribute to metastasis upon release. Future studies are warranted to gain more mechanistic insights on STAT3 stability in ascites and tumour microenvironment.

### 3.4 ISG15 and STAT3 modulate the exo‐ and endocytosis in ovarian cancer Ascites cells

Additionally, we show that activation of STAT3 (Figure 3b,c: Lane‐5) by IL6 and inhibition of ISG15 by a small molecule inhibitor, DAP5 (Figure 3b,c: Lane‐6), along with ISG15 overexpression (Figure 3b,c: Lane‐7) and knockdown (Figure 3b,c: Lane‐8) modulate the exo‐ and endocytosis of ascites derived TR127 cells. Our results demonstrate that ISG15 expression is directly proportional to the proteins being recycled (Figure S8c)**;** specifically, that exocytosis is being significantly reduced in TR127‐ISG‐KD cells (Figure 3d). The co‐localization of STAT3 in TSG101 positive vesicles, as observed by confocal microscopy in TR127 cells (Figure 3e), suggest that ISGylation of STAT3 enables them to be recognized as cargo to be packed into the vesicles for release in ascites. This supports our previous results that shows the release of STAT3 via the vesicles (Dorayappan et al., 2018).

### 3.5 Determining the role of ISG15 expression in ovarian cancer progression and metastasis

**FIGURE 4:**
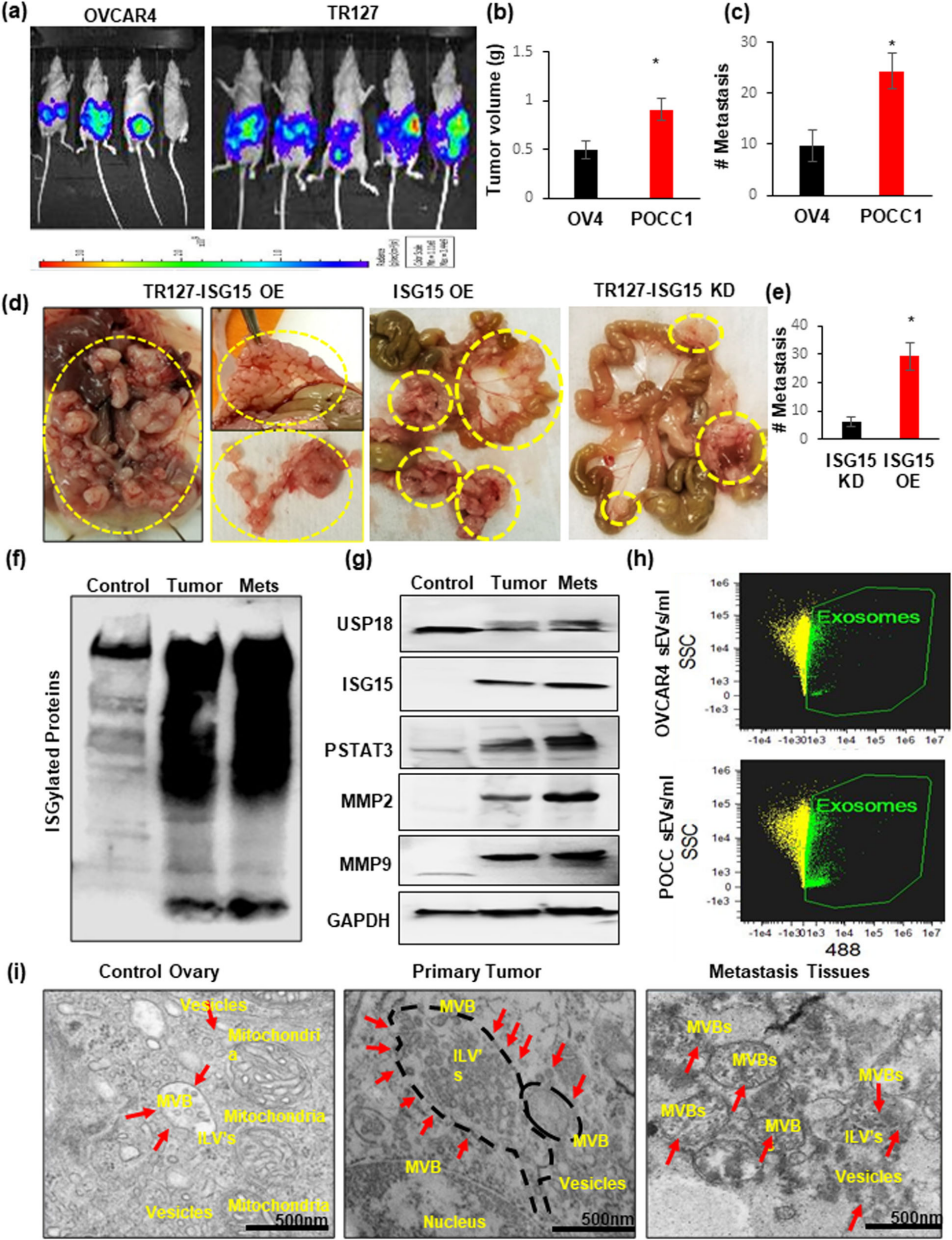
ISG15 overexpression of HGSOC ascites cells increased ovarian tumour progression and metastasis: (a) Luciferase stable transfected OVCAR4 or TR127 (1 × 10^5^ cells) were injected into the ovarian bursa in nude mice; (b and c) Tumour volume and metastasis analysed at week 5 in both groups (*n* = 5 mice ± SD). (d and e) HGSOC progression and metastasis observed in nude mice after intraperitoneal injections of ISG‐OE and ‐KD TR127 (2 × 10^6^ cells) (*n* = 4 mice ± SD). (f and g) ISG15 with ISGylation products, USP18 and downstream target proteins STAT3, MMP2 and MMP9, expression analysed in control ovary, primary and metastatic ovarian tumour tissues in mice (*n* = 5 mice ± SD). (h) Extracellular vesicle quantification by image stream flow cytometry in ascites developed in OVCAR4 and TR127 injected mice (*n* = 3 samples ± SD). (i) Increased sEV formation observed by Transmission Electron Microscopy (TEM) in primary and metastatic mice ovarian tumour tissues when compared to control ovarian tissue.

To elucidate the aggressiveness of ascites derived primary ovarian cancer cells, we developed an orthotopic ovarian cancer model in nude mice by injecting Luciferase (Luc) stable transfected OVCAR4 cells, which are less aggressive, and ascites derived immortalized POCCs‐TR127 into the ovarian bursa. The mice injected with TR127‐Luc cells had significantly higher tumour volume and more metastasis when compared to the mice injected with OVCAR4‐Luc‐cells (Figure 4a–c). Additionally, increased tumour metastasis were observed in an intraperitoneal (IP) mouse model when injected with TR127‐ISG‐OE when compared to ISG‐KD cells (Figure 4d,e).

The primary and metastatic tumour tissues from these mice had very high levels of ISG15 and its ISGylation products (Figure 4f). The downstream proteins responsible for tumour progression were also significantly elevated in the tumour and metastatic tissue (Figure 4g). Vesicles’ quantification from the mice revealed increased sEV in POCC ascites when compared to that of OVCAR4 ascites (Figure 4h). TEM studies on the mice tissue confirmed the presence of increased vesicles and MVBs in the primary and metastatic ovarian tumour tissue (Figure 4i, Figures S9 and S10).

This data suggests ISG15 contributes to ovarian cancer metastasis and progression via vesicle release as both a signaling mechanism and transportation of oncogenic cargo like STAT3. These results encouraged us to investigate the effects of EV inhibitor therapy using a relevant, pre‐clinical orthotopic tumour model.

### 3.6 Extracellular vesicles’ inhibition as a therapeutic strategy to reduce tumour progression and metastasis in ovarian cancer

**FIGURE 5:**
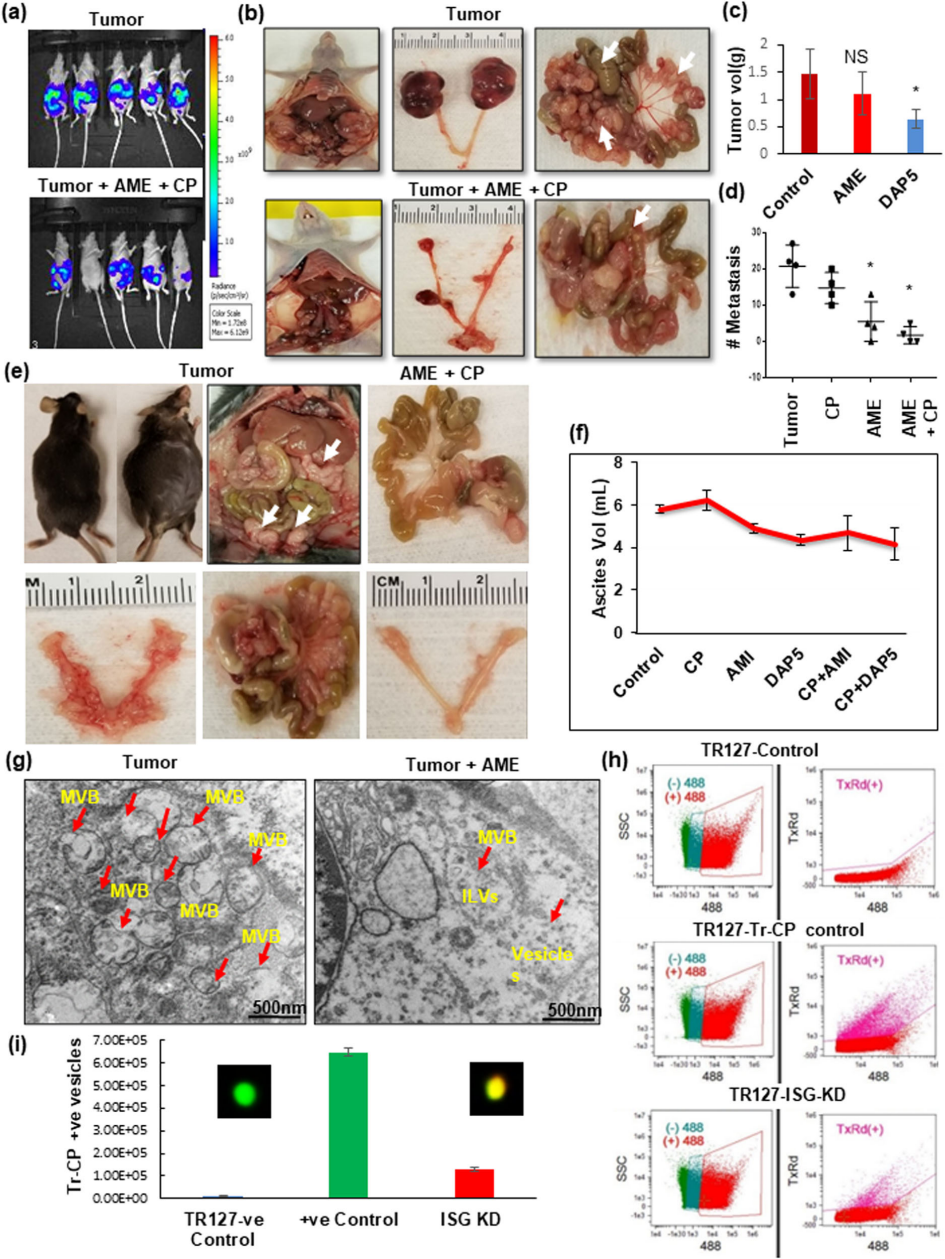
Blocking sEV and inhibition of ISG15 reduces ovarian tumour progression and metastasis. (a–d) Significant reduction in tumour growth and metastasis was observed in DAP5 (100 ppm in animal feed) treatment (ISG15 inhibitor) and in amiloride (AME)+ carboplatin (CP‐ 2 mg/kg b.wt.) treated group in orthotopic nude mice injected with luciferase stable transfected TR127 cells when compared to treatment with sEV inhibitor (AME‐ 2 mg/kg/ twice a week) alone for 5 weeks (*n* = 4 mice ± SD). (e and f) Tumour progression in immunocompetent mouse models injected orthotopically with immortalized ID8 cells, (1 × 10^6^ cells) treated with amiloride (2 mg/kg/twice a week) or DAP5‐100 ppm in combination with carboplatin (CP‐ 2 mg/kg b.wt., *n* = 3 mice ± SD). (g) sEV formation in amiloride treated mouse tissue compared to untreated tumour tissue as observed by TEM. (h and i) sEV quantification by image stream flow cytometry on POCC control and TR127‐ISG15‐Kd cells treated with Texas‐red labelled carboplatin (*n* = 3). The overlay of the Tr‐CP on the FITC+ve vesicle is shown in the inner square on the graph.

After observing that sEV and their cargo contribute to metastasis, we aimed to prevent the spread of disease using therapeutic strategies aiming to inhibit the vesicle release. We used both a known vesicle inhibitor, amiloride, as well as a small molecule inhibitor, DAP5, for targeting ISG15 along with conventional platinum‐based therapies (Figure 5a,b; Figure S11). For this study, TR127‐Luc‐cells were orthotopically injected into athymic nude mice to monitor the spread of cancer. Disease distribution was evaluated before and after treatment. We found that tumour volume and metastasis were significantly decreased in both the DAP5 alone treated group and DAP5 in combination with carboplatin (Figure 5c,d). A similar study was done in immunocompetent mice with mice ovarian cancer cells (ID8). The Ascites volume was significantly reduced when treated with amiloride and DAP5 in combination with carboplatin in two separate groups (Figure 5e,f; Figure S12). Our results from Figure 5a–f demonstrate that carboplatin alone was not effective, whereas the combination treatment resulted in a significant reduction in tumour growth without affecting body weight (Figure S13). Further, the TEM images of the mice ovarian tumour tissues revealed a significant reduction in vesicle formation in mice treated with amiloride (Figure 5g).

We further analysed if ISG15 expression could contribute to platinum sensitivity of the TR127 cells by quantifying the amount of Texas‐red labelled Cisplatin (Tr‐CP) positive vesicles released after treatment for 24 h. We found that TR127‐ISG‐KD cells had significantly lower vesicle counts than the TR127 control cells and therefore showed decreased Tr‐CP cellular efflux via the vesicles as captured and analysed by image stream analysis (Figure 5h,i; Figure S14a), suggesting that ISG15 expression contributes to cisplatin sensitivity. Additionally, the increased accumulation of Tr‐CP within the TR127 cells was confirmed by confocal microscopy after treatment with either amiloride or DAP5 (Figure S14b). These results strongly suggest the efficacy of amiloride and DAP5 targeting the vesicles and inhibiting the ISG15 in ovarian cancer treatment either alone or in combination with platinum therapy.

### 3.7 Identifying vesicular ISG15 as prognostic marker in ovarian cancer

**FIGURE 6:**
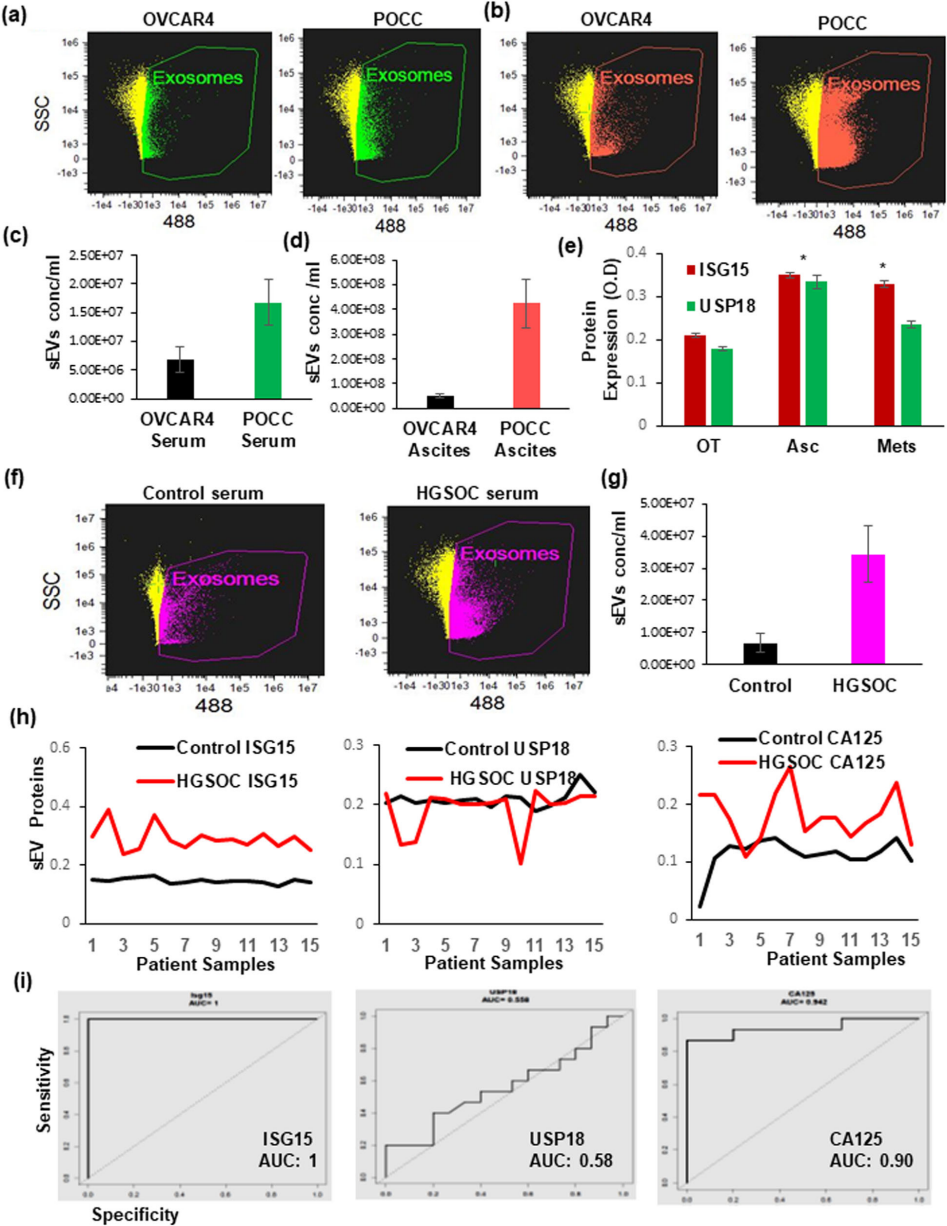
ISG15 expression in ascites and serum as prognostic biomarker in HGSOC mouse models and patients. (a–c) sEV quantification in OVCAR4 and TR127 transplanted mice serum and ascites (*n* = 3). (d) Relative expression of ISG15 and USP18 in primary and metastatic mouse ovarian cancer tissue compared to ascites by ELISA (*n* = 6). (e and f) sEV levels in control and HGSOC patient serum samples (*n* = 3). (g) ISG15, USP18 and CA125 expression in healthy control and HGSOC patient serum sEV (*n* = 15). (h) ROC curves based on ELISA results from small EVs isolated from controls and HGSOC serum samples analysed the ISG15, USP 18 and CA125 (*n* = 12).

Our previous work has identified various extracellular vesicular (EV) proteins that are elevated in early‐stage ovarian cancer (Dorayappan et al., 2019). Recent studies show that sEV can be used as a potential source of biomarkers in various diseases (Ciferri et al., 2021; Kugeratski et al., 2021) . In this study, we identified expression of vesicular ISG15 and USP18 represents a novel development in prognostic biomarkers for HGSOC patients. Image Stream Analysis (ISA) on vesicles isolated from the serum and ascites of mice injected with OVCAR4 cells and TR127 cells demonstrated significantly increased vesicles in both the serum and ascites of the TR127 mice (Figure 6a–c). The expression of both ISG15 and USP18 were significantly increased in the primary and metastatic tumour when compared with control mice ovarian tissue as analysed by ELISA (Figure 6d). Likewise, the vesicle concentration in the serum of patients with HGSOC was significantly elevated when compared with healthy control samples (Figure 6e,f). On analysing the comparative expression of EV‐ ISG15, USP18 and CA125 (Figure 6g,h) in advanced HGSOC patient serum and the whole serum (Figure S15), we found that vesicular ISG15 and CA125 expression were significantly elevated when compared to healthy subjects. Based on the statistical significance and AUC, vesicular ISG15 expression strongly correlates with HGSOC progression and metastasis. Therefore, ISG15 not only represents a potential therapeutic marker, but a promising prognostic biomarker as well. Further work is needed to validate these findings.

## 4 DISCUSSION

In the present study, we have shown the following novel findings: (i) Increased expression of ISG15 in ovarian cancer ascites and metastatic tissue; (ii) ISG15 *knockdown* inhibits sEV secretion and reduces the exocytosis of oncogenic cargoes resulting in reduced tumour growth and metastatic potential in vitro and in vivo, suggesting that ISG15 is required for sEV secretion and ovarian cancer metastasis; (iii) the sEV blocker, amiloride and ISG15 inhibitor, DAP5, significantly suppress ovarian cancer tumour growth and metastasis in an orthotopic tumour as well as immunocompetent OC mice models; (iv) Vesicular ISG15 and USP18 expression were found to be significantly increased in ovarian cancer ascites, serum and metastatic tissue, suggesting that it may serve as a potential biomarker.

Aberrant expression of ISG15 has been demonstrated in most human malignancies, including ovarian cancer, suggesting it has a pro‐tumour function (Burks et al., 2014; Desai, 2015; Desai et al., 2006; Li et al., 2014). Expression of ISG15 also contributes to malignant transformation of cells in culture (Desai et al., 2006; Desai et al., 2012). Recently, it has been gaining scientific thrust because of its newly recognized function of inhibiting the canonical ubiquitin pathway. However, very little information is available on the expression levels of the free ISG15 protein and its conjugates in human cancers. Recent literature demonstrates differing and mixed expression levels of free ISG15 and ISG15 conjugates in various human tumours (Alcala et al., 2020). As such, the correlation between ISG15 levels and cancer activity is currently unclear and likely depends on the cellular and the tumoral context. Based on our study results demonstrating elevated free ISG15 expression in HGSOC ascites, ascites derived vesicles and metastatic tissue, we hypothesized that ISG15 is a contributing factor in HGSOC progression and metastasis through sEV secretion. A previous study similarly showed that ISG15 expression levels in hepatocellular carcinoma tissues were significantly correlated with tumour volume, advanced stage, and cellular differentiation (Zuo et al., 2016).

It has previously been shown that ISGylation and ISG15 conjugation regulate sEV secretion in cancer cells (Han et al., 2018; Villarroya‐Beltri et al., 2016). However, it has been unclear how ISG15 contributes to increased sEV secretion and how this, combined with the release of vesicular oncogenic proteins, impact the ovarian cancer progression and metastasis. A recent study identified that increased ISGylation reduced the number of MVBs and impaired sEV secretion in Jurkat T cells (Villarroya‐Beltri *et al.*, 2016; Villarroya‐Beltri et al., 2017). Significantly, we have identified that ISGylation is increased in HGSOC ascites cells resulting in increased MVB formation and increased sEV secretion, suggesting that ISG15 activity may be dependent on cell type and/or microenvironment.

Specifically in tumorigenesis, the roles of free ISG15 and ISGylation are not well‐defined, despite many studies. Some studies suggest that protein ISGylation is an antagonistic system to the ubiquitination system in order to provide stability to proteins in breast cancer cells (Pinto‐Fernandez et al., 2021; Tecalco‐Cruz et al., 2022). Concurrent to the above findings, we have shown that ISGylation of STAT3 in ascites cells may be contributing to its stability and increasing the sEV release. Conversely, protein ISGylation has also been associated with the destabilization of proteins, such as cyclin D1 and p53 (Guo et al., 2012; Huang et al., 2014). Thus, free or conjugated forms of ISG15 have a complex role in tumorigenesis in a wide range of human tumours and cancer cell lines. In our current study, we have observed very high sEV secretion in serum and ascites of mice injected with immortalized patient ascites derived primary ovarian cancer cells (POCC) cells when compared with less aggressive HGSOC cells‐OVCAR4. In addition, the expression of ISG15 and ISGylation in metastasis tissue were significantly increased than in the primary ovarian cancer tissue of our orthotopic mice model studies. This elevated expression of ISG15 and ISGylation resulted in the release of high levels of vesicles with oncogenic proteins contributing to aggressive tumour progression and metastasis. The effect of ISGylation of specific protein targets in the tumour microenvironment will also be the focus of our further research.

Previous studies have shown that increased USP18 expression promotes tumorigenesis by increasing stability of critical ISG15‐conjugated oncogenic proteins (Basters *et al.*, 2017; Liu et al., 2020; Pinto‐Fernandez *et al.*, 2021). In our study, we have shown that ISG15, ISGylation and USP18 are increased in ascites cells when compared with normal ovarian surface epithelial cells and less aggressive high grade serous ovarian cancer cells. However, the oncogenic or tumour suppressive outcome of the USP18 varies based on the differing cellular and tumour contexts (Kang & Jeon, 2020; Liu et al., 2020). In our previous work, we have shown that STAT3, a major oncoprotein in ovarian cancer, is highly activated in the ascites (Saini *et al.*, 2017; Saini et al., 2018). Here, we show that STAT3 is also a target of ISGylation in ascites cells. We suspect this may be the contributing factor to its constitutive activation, although further studies are warranted.

Recent studies have shown that targeting ISG15, using knockdown or a small molecule inhibitor, significantly inhibits cancer cell proliferation and induces apoptosis by increasing caspase‐3 and caspase‐9 (Kariri *et al.*, 2021; Li *et al.*, 2014; Sainz *et al.*, 2014). Further, blocking sEV release enhances cisplatin (CP) uptake leading to enhanced cytotoxic effects in cancer cells (Kamerkar et al., 2017; Rojas et al., 2017; Rowson‐Hodel et al., 2016). Amiloride (AME) is a potassium‐sparing diuretic approved by the FDA for the treatment of hypertension and is also known to inhibit the release of vesicles. AME has been shown to have an anti‐proliferative and synergistic effect in combination with cisplatin in cancer cells (Dorayappan *et al.*, 2018; Rojas *et al.*, 2017; Rowson‐Hodel *et al.*, 2016; Tang et al., 2013). Our current study demonstrates that inhibiting ISG15 using the small molecule inhibitor, DAP5, and inhibiting sEV release using AME reduces sEV secretion and prevents CP efflux in HGSOC cells. Our previous report and the current results show that chemoresistant OC cells are highly sensitive to CP following pre‐treatment with AME, making this drug a potential re‐purposed therapeutic candidate for chemoresistant OC. This result is consistent with a published study showing that treatment with norcantharidin on ISG15 knockdown cells significantly increased apoptosis compared to ISG15 KD or norcantharidin alone treatment in hepatic cancer (Chen et al., 2019).

Our study has demonstrated that the augmented release of vesicles from ascites cells and the presence of ISG15 within these vesicles can serve as potential biomarkers for evaluating clinical prognosis in ovarian cancer (Guo et al., 2018; Kalluri & LeBleu, 2020; Liang et al., 2021). Similarly, a previous report showed that vesicular protein could detect early pancreatic cancer (Melo et al., 2015), and ISG15 expression was increased in hepatic cancer, suggesting that it may serve as a potential prognostic marker in tumour biomarker for drug sensitivity (Chen et al., 2016; Desai et al., 2008; Kariri *et al.*, 2021). In the present study, vesicular ISG15 expression was found to be significantly increased in HGSOC ascites, serum and metastatic tissue. Further research is necessary to validate the clinicopathological significance of vesicular ISG15 levels in patients with HGSOC and their correlate with disease progression, recurrence and/or platinum resistance.

In conclusion, our study demonstrates an increased expression of ISG15 in HGSOC ascites. Our findings suggest that ISG15 may drive the progression and metastasis of HGSOC and serve as a potential biomarker for ovarian cancer prognosis. Furthermore, our results indicate that knockdown of ISG15 or blocking sEV secretion effectively inhibits HGSOC growth and metastasis. These novel insights into the role of ISG15 in HGSOC provide valuable information for the development of molecular targeted treatments and monitoring of HGSOC. Future clinical translation of our findings is warranted and may lead to significant improvements in the diagnosis and management of HGSOC.

## AUTHOR CONTRIBUTIONS

Kalpana Deepa Priya Dorayappan, Vincent Wagner and Dongju Park conducted experiments, analysed the data, wrote the paper and were involved in the preparation of figures. Wafa Khadraoui and Michelle D.S. Lightfoot helped in identifying the patient sample from CCC used in the current study and helped in editing the manuscript. Dongju Park, Meghan M. Newcomer and Deepika Kalaiyarasan helped with in‐vitro and in‐vivo mice model studies. Takahiko Sakaue and Lianbo Yu provided support with statistical analysis. G Larry Maxwell provided the patient serum samples and support for the study. Qi‐En Wang, David O'Malley and Raphael E. Pollock provided resources for cell lines and serum samples and division research funding support to mice studies. David E. Cohn and Karuppaiyah Selvendiran designed the experiments, analysed the data, wrote the manuscript, provided support for the study and preparation of figures.

## CONFLICT OF INTEREST STATEMENT

All authors declare that there are no conflicts of interest.

## Supporting information

Supporting Information

Supporting Information
