# Supplementary material for: ISG15 mediates the function of extracellular vesicles in promoting ovarian cancer progression and metastasis
Source: J Extracell Biol. 2024 Jan 31;3(2):e92. doi: 10.1002/jex2.92 (PMC11080709; doi:10.1002/jex2.92)
Supplement: Supplementary file 1 — Supporting Information [file JEX2-3-e92-s001.docx]

**Materials & Methods:**

**Cell culture conditions:**

All human Immortalised ovarian surface epithelial cells (OSE 385 and 386) used in the current study named as OSE 1and 2 , were maintained in OSE medium composed of 50:50 medium 199: MCDB105 (Sigma) supplemented with 10% fetal bovine serum (FBS), 25mM HEPES and 100units/mL Penicillin/Streptomycin. High Grade serous ovarian cancer (HGSOC) cell lines (OVCAR3 and -4), and ascites derived primary ovarian cancer cell lines (POCCs) used in this study were maintained in 75cm^2^ tissue culture flasks in RPMI- 1640 culture media (Thermo Scientific) supplemented with 10% fetal bovine serum (FBS) and small extracellular vesicles ((sEV)‐depleted FBS for sEV isolation studies), 1mM sodium pyruvate, 0.1mM nonessential amino acids and 100units/mL penicillin‐streptomycin. All cell cultures were maintained in a humidified incubator at 37°C with 5% CO2.

**Immunoblotting**:

Cells were washed twice with PBS and lysed with lysis buffer containing proteases at 4°C for 45 min. The lysates were centrifuged at 14,000 rpm for 15 min, and the total protein concentration in the supernatants were determined using BCA kit from Thermo Fisher Scientific (Waltham, MA). Then the protein samples were subjected to SDS-PAGE and transferred onto PVDF membranes (Bio-Rad). Membranes were blocked for 1 h in 5% nonfat milk powder (w/v) in TBST and incubated overnight at 4°C with primary antibody diluted in TBST containing 5% nonfat milk powder (w/v). After three washes with TBST, membranes were incubated for 1 h with horseradish peroxidase-conjugated secondary antibodies (1:10,000 dilution) in TBST containing 5% nonfat milk powder (w/v). The immunoblots (or western blots) were developed using enhanced chemiluminescence-detection system (ECL Advanced kit; Amersham Biosciences) and imaged on a Li-COR machine and analyzed by Image Studio software.

**Isolation of Vesicles from different biological samples:**

To isolate tumor extracellular vesicles (SEV) from culture media, the immortalized ovarian cancer cells were cultured for two days (48hr) in medium with sEV depleted FBS (Purchased from System Bioscience, CA, US). The conditioned medium was centrifuged at 400×g for ten minutes to remove whole cells and this supernatant was then centrifuged at 10,000×g for 30 minutes to remove large microvesicles and apoptotic vesicles. The supernatants were filtered through 0.22 μm porous membranes, followed by ultracentrifugation at 100,000g for 1hr at 4°C to isolate the small extracellular vesicles (sEVs). The EV pellet was then washed in 10 ml 1× PBS followed by repeated ultracentrifugation at 100,000g at 4°C for 1hr. The final pellet was re-suspended in PBS and the vesicular protein concentration was determined using the micro BCA protein assay from Thermo Fisher Scientific (Waltham, MA) according to manufacturer’s instructions. The serum used in the current study was comprised of frozen samples and ascites were obtained fresh from a population of patients undergoing surgery as approved based on the Ohio State University IRB (Study number 2004C0124). For isolation of vesicles from 250 μl of cell-free serum and ascites, samples were thawed on ice. Samples were diluted in 2.5ml of of 1X PBS and filtered through a 0.2 μm pore filter and proceeded with the centrifugation steps similar to the culture medium protocol. The resulting EV pellet was re-suspended in 100μl of cold PBS or lysis buffer according to the downstream applications such as Image stream flow cytometry, ELISA and Western blotting as previously described (Dorayappan et al. 2018 Oncogene, 2022 Cancer Research)

**Confocal microscopy:**

Normal OSE cells and POCCs were grown on cover-slips in a 6 well plate filled with the appropriate conditioned culture medium. When cells reached the desired confluence, equal volume of the lyso-tracker-red dye-working solution was added and incubated in a 37 °C, 5% CO2 incubator for 30 minutes. The cells were washed twice with pre-warmed PBS, and then fixed with 4% paraformaldehyde. Lysosome localization was then imaged with confocal microscopy.

**Image stream Flow cytometry:**

The Image Stream flow cytometer was used to quantify the vesicles in different samples.

Fluorescent markers are required for Image Stream analysis due to the low level of sEV scattering, and their brightfield (BF) images being below the resolution of the BF camera for the majority of sEV. CFSE, a fluorescent cell-permeable dye that covalently binds through its succinimidyl group to intracellular proteins at their lysine residues and other amine sources was utilized. The Image Stream was equipped with a 60X objective to detect particles in the EV size range, with a numerical aperture (NA) of 0.9 and a resolution of 0.3 μm^2^ per pixel. Although many of the vesicles are below the size of the pixel resolution, when labelled with fluorescent molecules these smaller vesicles become detectable due to the intensity of the signal, and the width of the core stream is reduced to 7μm to increase the frequency of in-focus objects. The speed was set to the lowest setting for maximum resolution. All sheath buffers were filtered with 0.1μm filters to ensure that there were minimal background particulates. The instrument was equipped with 488 nm and 561nm lasers to be compatible with the CFSE and Texas red, and 200 mW was used to increase the number of photons generated per fluorochrome molecule, including the 758 nm laser for scatter measurements. Based on the high scatter intensity of the PSB, a collection gate was established that eliminated the beads from the final measurement. The data analysis of the vesicle concentrations were done using measurements were done Image Stream Data Exploration and Analysis Software (IDEAS R 6.2 EMD). All samples were collected using the INSPIRER instrument acquisition software. Samples were run in the following order to make sure there was no carryover of any fluorophores from sample to sample: 1) buffer only, 2) unstained sEV, 3) buffer plus dye, and 4) samples. The Poly styrene beads (PSB) was used as a reference gate to identify the scatter of the vesicle particles.

**ISG15-Overexpression (OE) and knockdown (KD) studies:**

For upregulation of ISG15 expression in ascites derived POCC transfection ready ISG15 expression plasmid with ISG15 (NM_005101) Human Tagged ORF Clone (Cat#RG201235) containing the overexpression protein construct cloned into the pCMV6-AC-GFP, mammalian vector with C-terminal tGFP tag was used with Turbofectin transfection reagent . The mammalian cell selection with Neomycin resistance was used to confirm the protein overexpression by fluorescence microscopy. Likewise for transient downregulation of ISG15 expression in POCCs, Human ON-TARGET plus siRNA targeting ISG15 (Cat# J-004235-21-0050) and ON-TARGET plus Non-targeting Control siRNA (catalog# D-001810-01-05) were used from from Dharmacon™ siRNA solutions (Horizon discovery biosciences, USA) with DharmaFECT 1 transfection reagent (Dharmacon) according to manufacturer’s instructions. The silenced For a stable knockdown of ISG15 in POCCs, shRNA plasmid with four unique 29mer shRNA constructs in retroviral untagged vector from ORIGENE (NM_005101) )was used alongside a negative scrambled shRNA for control according to manufacturer’s protocol.. . After puromycin selection (1 μg/ml), the TR127-ISG-Kd pooled stable clones were selected and used in the *in-vivo* pre-clinical mice model studies.

**Cell surface biotinylation for protein trafficking:**

Cultured POCCs were cooled to 4°C on ice maintaining a temperature restrictive to endocytosis. The membrane impermeable sulfo-NHS-SS-biotin reagent was added, and cells were incubated in the dark to allow sufficient time for biotin labels to covalently attach to the surface proteins. Cells were then removed from ice and incubated at 37°C for approximately 30 minutes to allow the endocytosis of biotinylated surface proteins. Following this, the cells were cooled to 4°C to stop the endocytosis and a potent reducing agent Tris-(2-carboxyethyl) phosphine hydrochloride) (TCEP-HCL) was added to release the biotin groups from labeled, un-endocytosed proteins. Next, cells were lysed to expose the biotinylated proteins. Following this, lysates were added to streptavidin-coated beads and biotinylated proteins allowed to bind. Beads were washed with cold phosphate buffered saline and eluted with the SDS sample buffer to denature bound proteins off beads and enable their recovery in the eluate. Proteins in the eluate are separated based on their molecular mass by gel electrophoresis. Lastly, Western blotting and probing of the blot with biotin was performed. Percentage endocytosed protein was quantified from the resulting band densities in Image Studio. To assess recycling, we performed a standard biotinylation protocol in one group of cells, with modifications such as re-heating to allow recycling of some of the internalized, biotin tagged proteins after stripping the biotin off the un-endocytosed surface proteins. Finally, by calculating the difference between the internalized proteins before and after recycling, we were able to quantify percent of proteins recycled back to the membrane.

**ISG15 Pull down Assay:**

A 500-μg sample of the total protein was used for immunoprecipitation from each sample. ISG15 antibody (10uG) was incubated with cell lysates for 12 h, followed by addition of 20 μl PureProteome Protein G Magnetic Bead System. The matrices were washed four times with the same lysis buffer. After being boiled for 8 min in the presence of 2-mercaptoethanol, samples containing cell lysate protein were separated on a 10% sodium dodecyl sulfate polyacrylamide gel (SDS-PAGE gel) and then transferred onto equilibrated PVDF membranes. After blocking with 5% nonfat milk powder (w/v), the membranes were incubated with the primary antibodies as described above. The bound ISG15 antibodies were detected with horseradish peroxidase (HRP)-conjugated secondary anti-mouse IgG using an enhanced chemiluminescence-detection system (ECL Advanced kit; Amersham Biosciences). The membranes were then probed for pSTAT3 and TSG101 antibodies.

**Vesicle labelling and internalization:**

50 μL of 10X Exo-Glow (System Biosciences) were added to 500 μL of re-suspended intact vesicles in 1X PBS and mixed well. The sEV suspension was then incubated at 37 °C for 10 minutes and placed on ice for 30 min, after which the samples were centrifuged using a column to remove the excess labelling. The labelled sEV were cultured with OVCAR-4 cancer cells and incubated for 24 hours and imaged after washing the cells twice with PBS on the Olympus FV1000 confocal microscope.

**Co-localization studies using Confocal microscopy:**

Co-localization of STAT3 and TSG101 was confirmed in another experiment by cell membrane permeabilization to probe for the internal proteins (STAT3 and TSG101) with primary antibodies and incubated overnight. After the overnight incubation the cells were washed with TBST-3X at RT and then probed with different fluorophore conjugated secondary antibodies. Nucleus was counterstained with DAPI and visualized by confocal microscopy. The images were collected with a FluoView 1000 laser scanning confocal microscope (Olympus, Center Valley, PA) using a 1.42 N.A., 40 and 60X, oil immersion objective.

**Orthotopic ovarian tumor and Intraperitoneal mice tumor model studies for metastasis:**

Nude mice and immunocompetent mice were used in the present study with different cell lines in two different tumor models. To demonstrate the role of ISG15 in ovarian tumor progression, the ISG-15 OE and KD POCCs (2× 10^6^ cells in 100μL of PBS) were introduced into the mouse peritoneum by intraperitoneal (i.p.) injections to observe the peritoneal metastasis in 6-week-old BALB/c nude mice obtained from the OSU transgenic mice core lab. Further, to demonstrate the efficacy of the sEV inhibitor- Amiloride (AME) and ISG15 inhibitor (DAP5), mice were orthotopically injected with the POCC- luc cells(1X10^5^ cells in 10uL sterile PBS) into the ovarian bursa of the immunocompetent mice and treated with amiloride and DAP5 (a small molecule inhibitor against ISG15) alone and in combination with cisplatin. *In-vivo* bioluminescence imaging was done periodically to confirm tumor growth. After sacrifice, the tumor weight and volume were measured.

**Statistical Analysis:**

Statistical Analysis Data are presented as mean±1 standard deviation. To determine statistical signiﬁcance among groups analysis of variance (ANOVA) was ﬁrst performed. If ANOVA was statistically signiﬁcant, the student’s t‐test was then performed to compare sub‐groups, p‐value <0.05 was considered as statistically signiﬁcant.
