# Supplementary material for: ISG15 mediates the function of extracellular vesicles in promoting ovarian cancer progression and metastasis
Source: J Extracell Biol. 2024 Jan 31;3(2):e92. doi: 10.1002/jex2.92 (PMC11080709; doi:10.1002/jex2.92)
Supplement: Supplementary file 2 — Supporting Information [file JEX2-3-e92-s002.pdf]

ISG15 mediates the function of extracellular vesicles in promoting ovarian cancer progression and metastasis

Kalpana Deepa Priya Dorayappan[1], Vincent Wagner[1], Dongju Park[2], Meghan M. Newcomer[1], Michelle D.S. Lightfoot[1], Deepika Kalaiyarasan[1], Takahiko Sakaue[1], Wafa Khadraoui[1], Casey Cosgrove[1], Qi-En Wang[3], Larry J. Maxwell[4], David O'Malley[1], Raphael E. Pollock[5], David E. Cohn[1] and Karuppaiyah Selvendiran[1.]

[1.] Division of Gynecologic Oncology; The James Comprehensive Cancer Center, Ohio State University, Columbus, Ohio.

[2] Molecular Genetics; The James Comprehensive Cancer Center, Ohio State University, Columbus, Ohio.

[3] Dept. of Radiation Oncology, The James Comprehensive Cancer Center, Ohio State University, Columbus, Ohio.

[4] Inova Schar Cancer Institute;

[5] Division of Surgical Oncology, The James Comprehensive Cancer Center, Ohio State University, Columbus, Ohio.

(a)

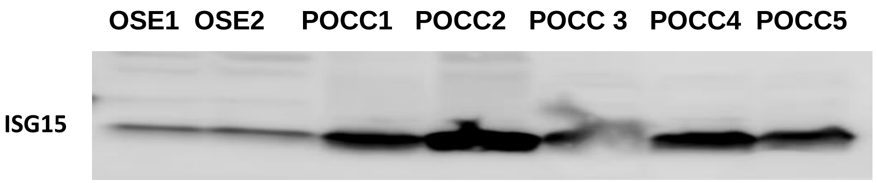

(b)

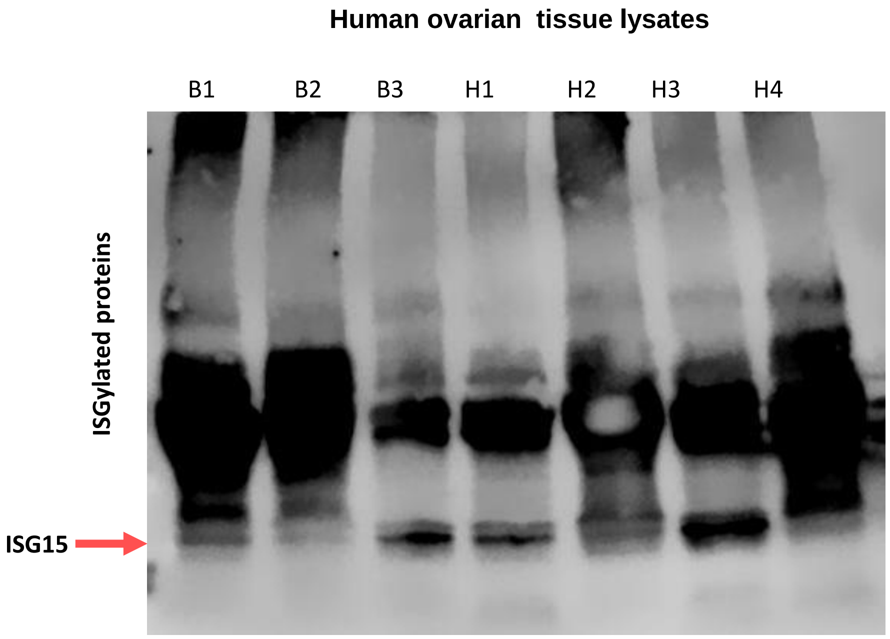

Supplementary Figure 1: a) Comparison of ISG15 expression in immortalized ovarian epithelial cells (OSE-385 & 386) and different patient ascites derived ovarian cancer cells (POCC) by Western blot. **b)** Comparison and ISG15 and Isgylation in Benign (B1-B3) and HGSOC (H1-H4) patient ovarian tumor tissues.

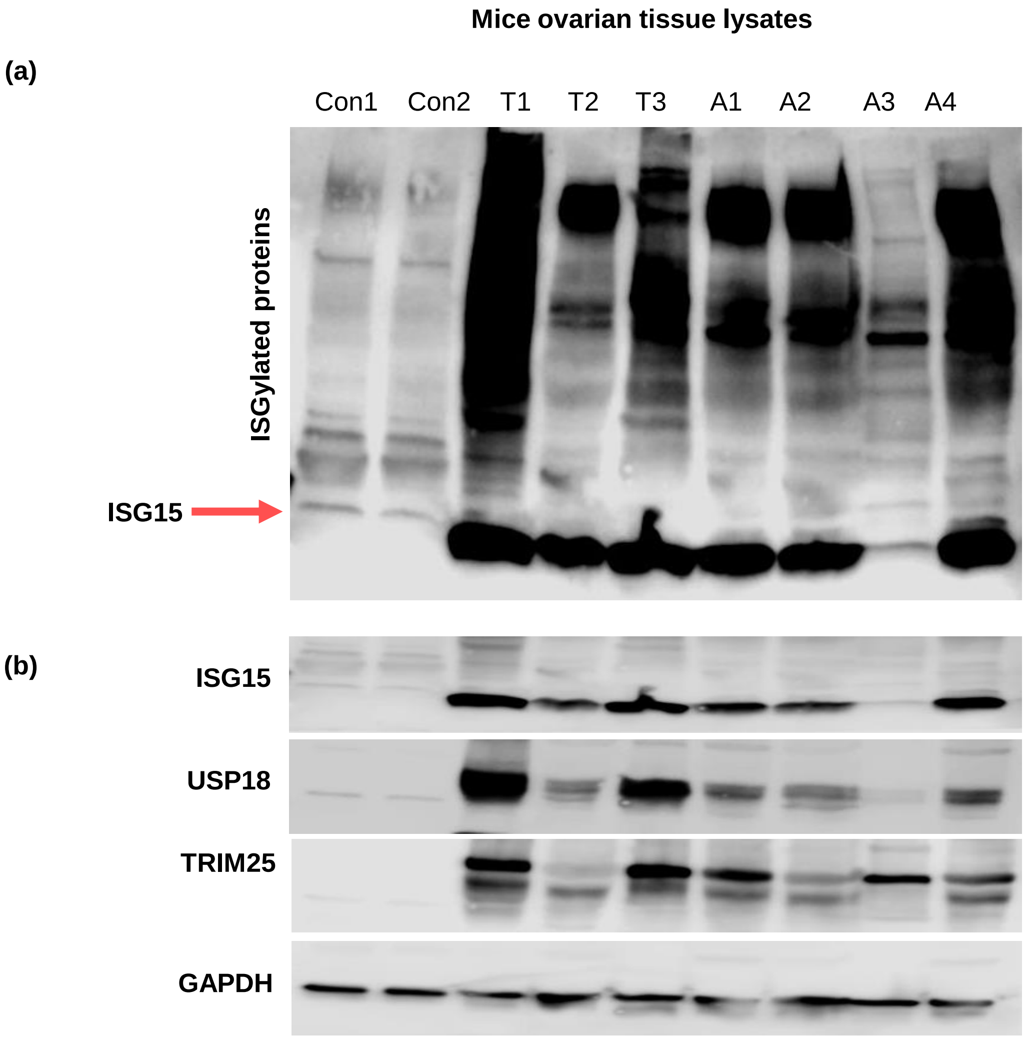

Supplementary Figure 2 a,b) Comparison of ISG15, USP18 and TRIM25 expression in mice ovarian control (Con1,2), orthotopic ovarian tumor tissues (T1-T3) along with the ascites samples (A1-A4) derived from POCC tumors in mice.

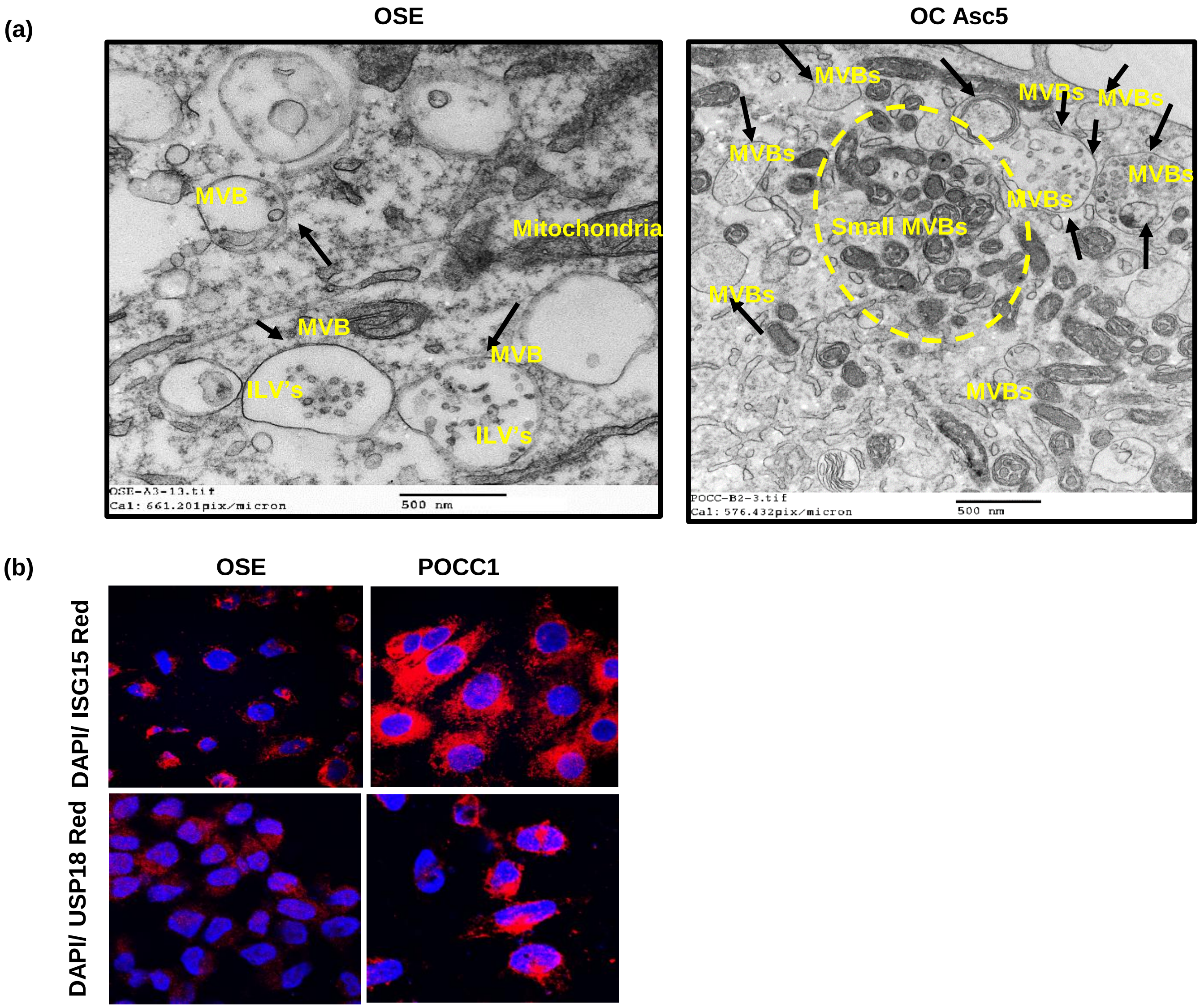

Supplementary Figure 3: a) Representative Transmission electron micrographs (TEM) of vesicles and multi vesicular bodies(MVB) with small intraluminal vesicles(ILVs) in OSE cells and ovarian cancer ascites cells (OC Asc5). Ascites cells show densely packed vesicles and increased vesicle numbers.

b) Increased ISG15 and USP18 expression in ascites derived HGSOC cells in comparison to OSE confirmed by confocal microscopy.

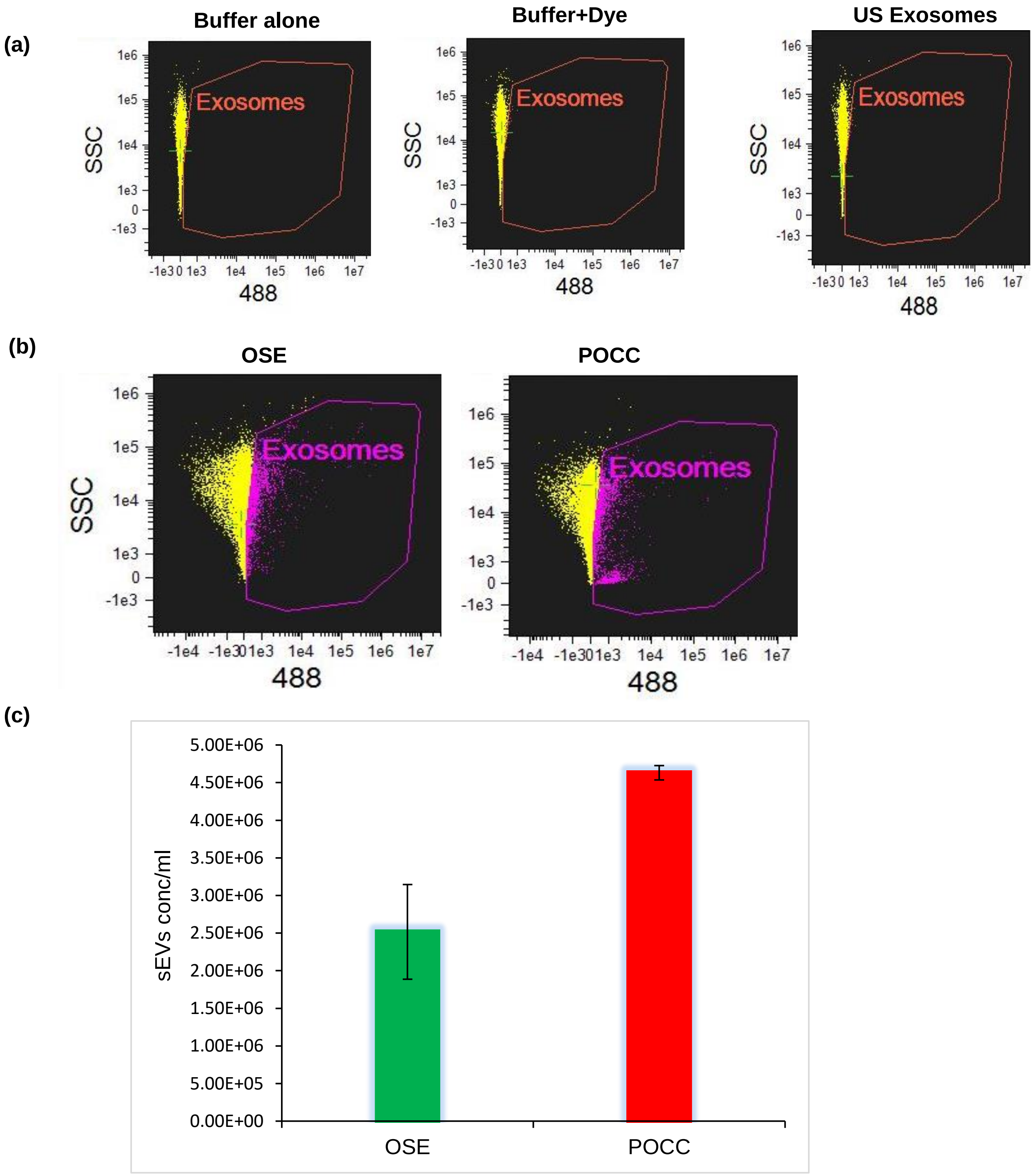

Supplementary Figure 4: a) Image stream analysis on controls: Buffer alone, buffer alone, Buffer+dye and Unstained exosomes. **b)** Image Stream flow cytometry analysis of vesicles released by normal ovarian surface epithelial cells (OSE) and patient ascites derived ovarian cancer cells (POCC) and c**)** the graph represents the objects (small Evs)/mL captured in the samples(n=3±SD)

Protein expression of ISG15 in POCC- ISG-OE and KD cells

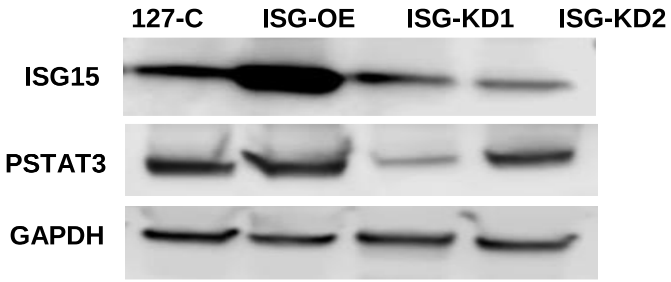

Supplementary Figure 5: Confirmation of overexpression and knockdown of ISG15 after 48hr transfection by western blot in ascites derived TR127 cells with the respective overexpression and knockdown plasmids.

Rab7+ve endosomes-(GFP labeled) show decreased endo- lysosomal fusion in TR127 – Control cells

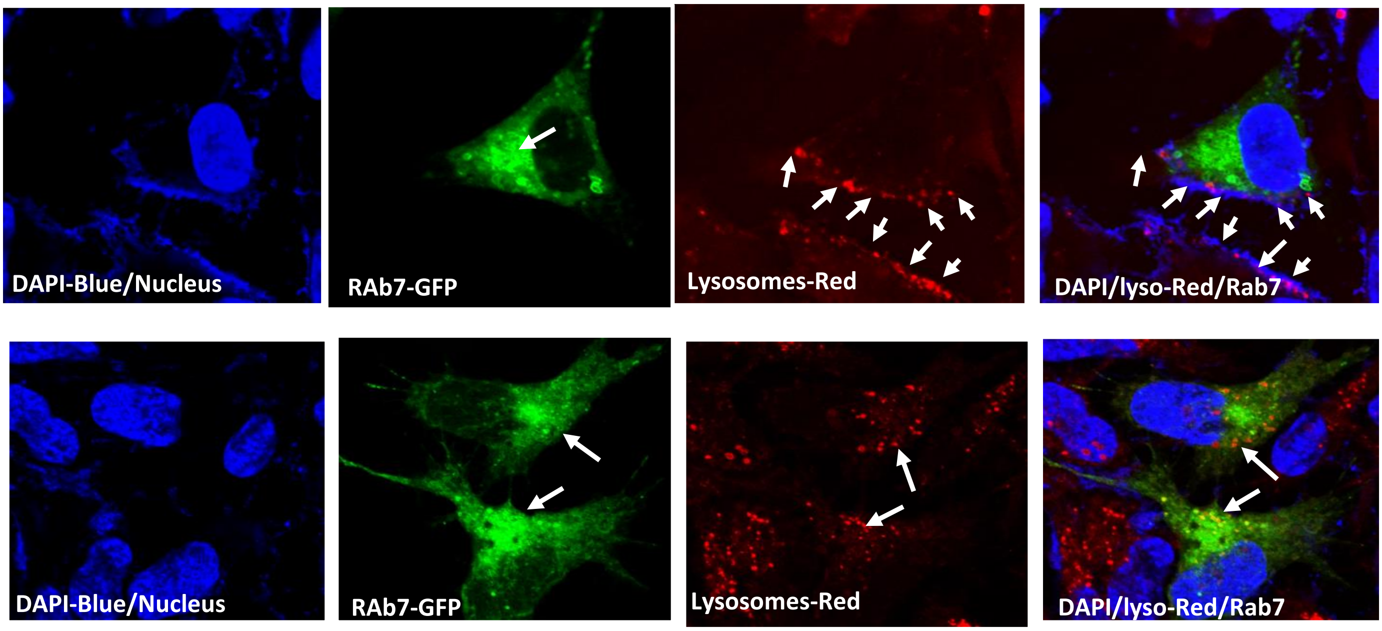

Rab7+ve endosomes(GFP labeled show increased endo- lysosomal fusion in TR127-ISG-KD cells

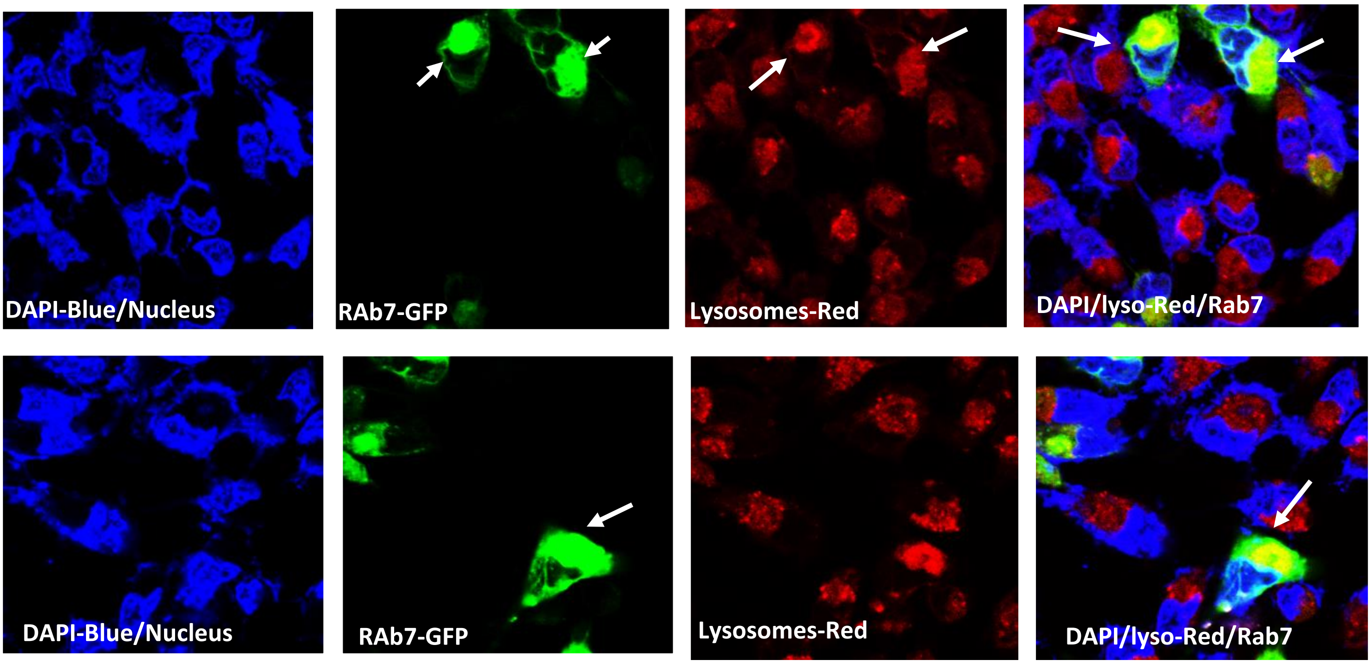

Supplementary Figure 6: Role of ISG15 in lysosomal degradation pathway- Ascites derived TR127 cells were transfected with ready-to-use fusion construct of Rab7a and emGFP (Cell Light™ Late Endosomes-GFP, BacMam 2.0), where it expresses GFP fused to Rab7. The late endosomes-(Rab7-GFP) along with lysosome tracker showing the dynamic cellular process of lysosomal fusion with Rab 7- GFP^{+ve} endosomes in POCC-ISG15kD cells when compared to POCC control cells by confocal microscopy(Mag-40X).

(a) **Exo internalization in HGSOC cell line-OVCAR4**

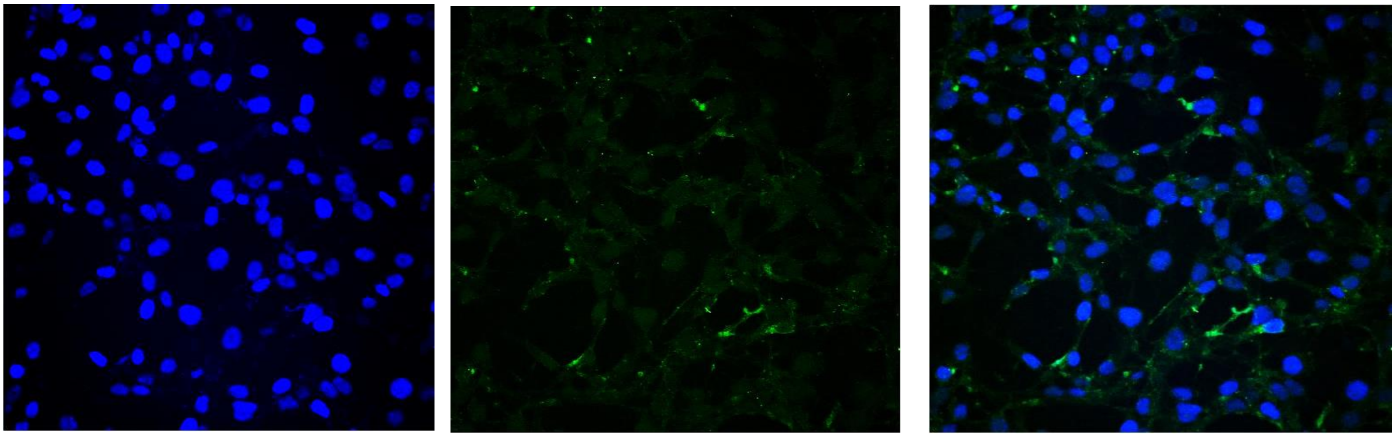

(b) **Confirmation of the uptake of GFP labeled ISG15- OE plasmid in POCC cells**

POCC- Vector control **POCC- ISG15-OE cells**

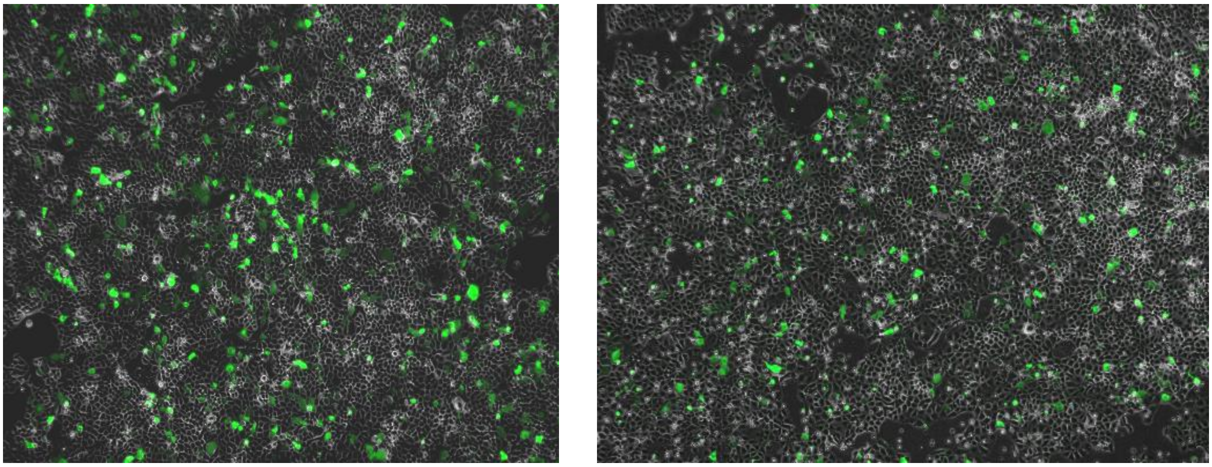

Supplementary Figure 7: a) Isolated vesicles from POCC- ISG15KD or overexpression cells were labeled with exo-glow-green, and co-cultured with Wt OVCAR4 cells in conditioned medium. Exosome internalization was confirmed after 24 hr incubation by confocal microscopy (n=3). **b)** Confirmation of uptake of pCMV6-AC-GFP with ISG15 overexpression protein construct after 48hr of transfection with Human Tagged ORF Clone alongside the control empty vector by fluorescence microscopy to assess the transfection efficiency using turbofectin DNA transfection reagent from ORIGENE.

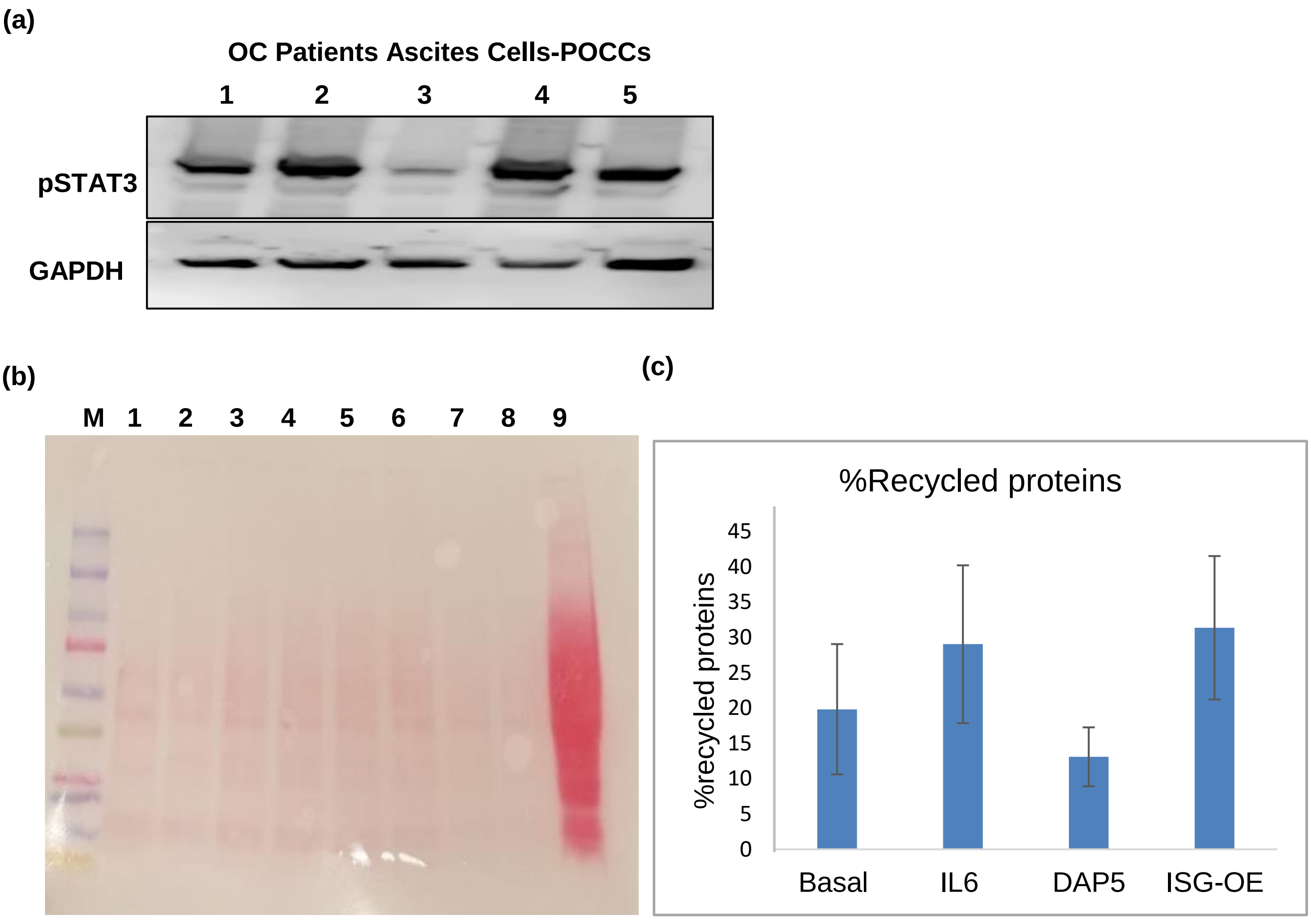

Supplemetary Figure 8: a) Expression of activated STAT3 in patient ascites cells. **b)** Ponceau staining on the PVDF membrane represents equal loading of proteins in all lanes in the ISG15 pull down assay. **c)** The graph represents the percentage of proteins recycled out to the surface of the cells by cell surface biotinylation assay in basal, STAT3 activated (IL6), ISG inhibited (DAP5) and OE cells to show the involvement of STAT3 and ISG15 in protein trafficking.

Mice control ovary and primary ovarian tumors

Control ovary **Primary ovarian tumors**

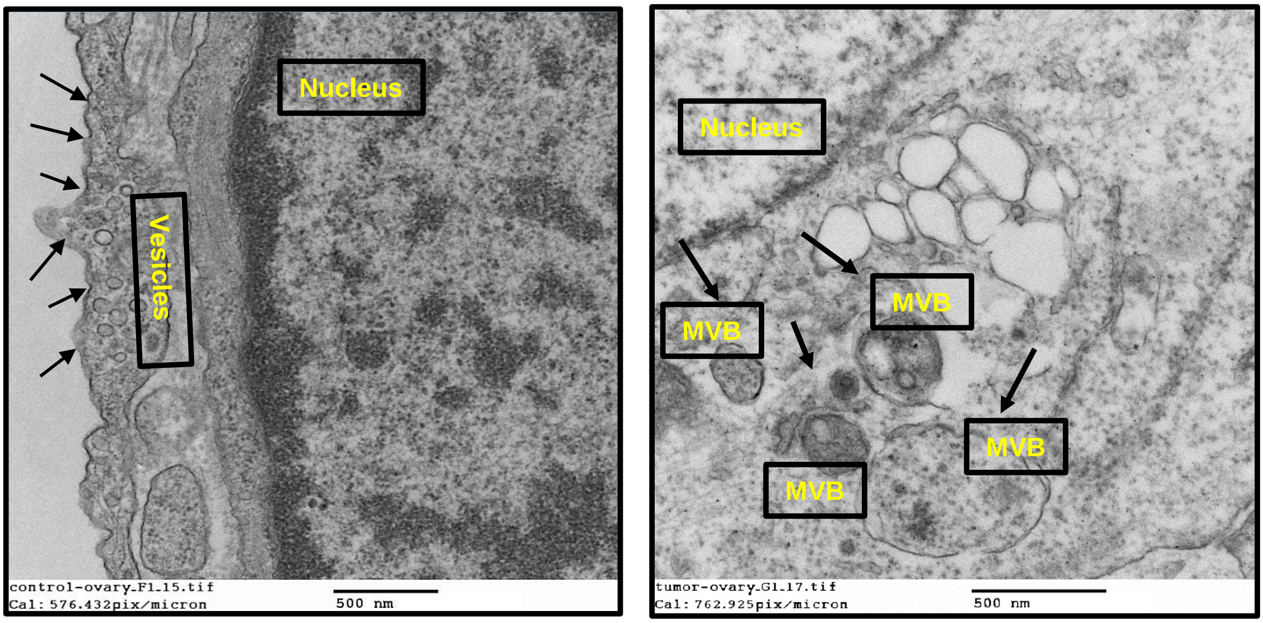

Supplementary Figure 9: Mice control and primary ovarian cancer tissues were prepared by chemical fixation and thin sectioning. TEM images show the clear architecture in the control ovary with fewer vesicles and MVBs as compared to the chaotic architecture in primary tumor tissues with increased number of vesicles and MVBs (Black arrows, scale bar-500nm). High magnification micrograph focuses on MVBs harboring smaller vesicles of different sizes and shapes (TEM was performed on three independent tumor tissues).

TEM images of ovarian tumor metastatic tissues

Mesentery metastasis

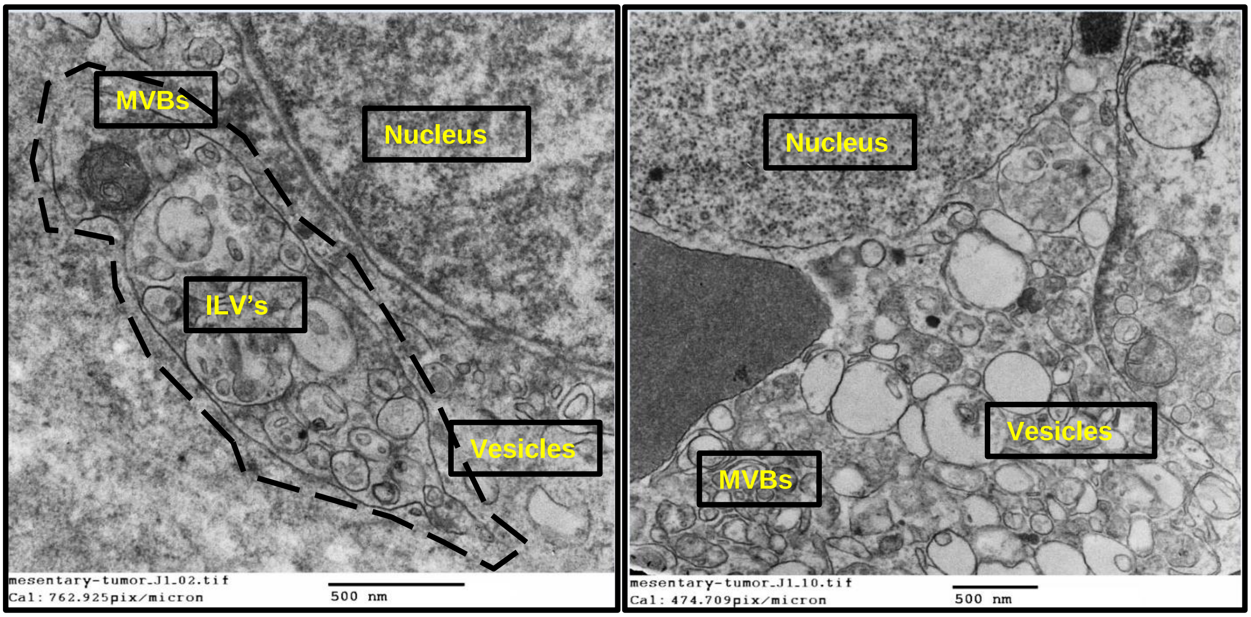

Diaphragm metastasis

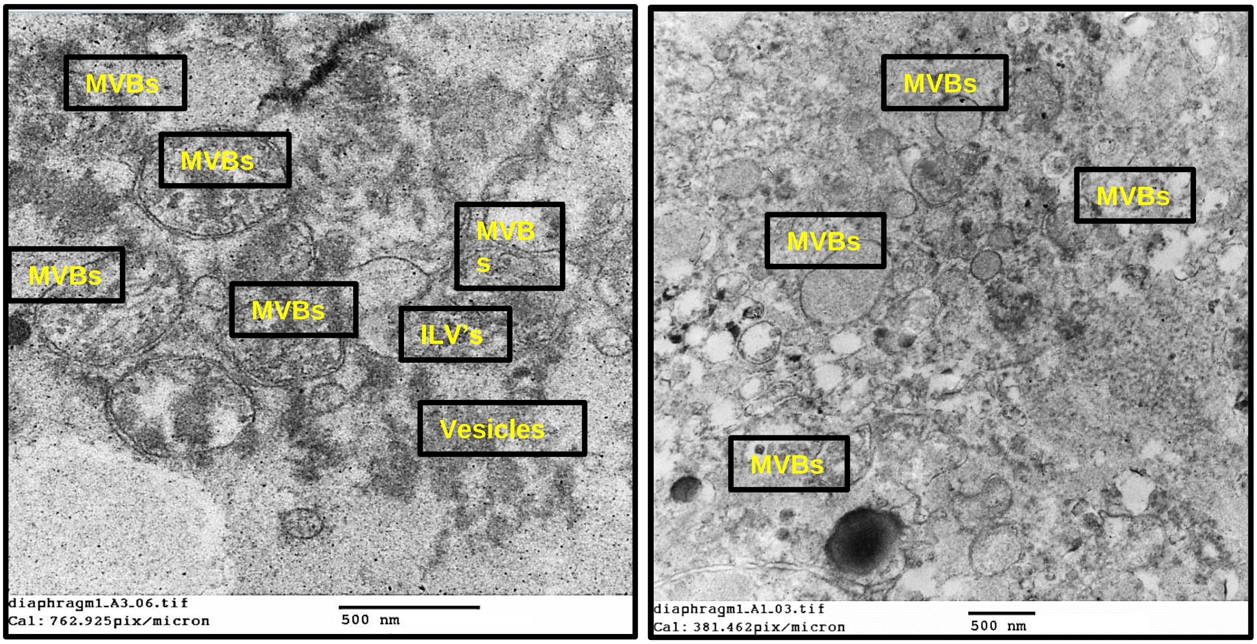

Supplementary Figure 10: Mice metastatic tissues (Mesentery and diaphragm) were prepared by chemical fixation and thin sectioning. TEM images of the ovarian tumor metastatic tissues mesentery and diaphragm with a chaotic architecture in tumor tissues with increased number of vesicles and MVBs (Black arrows-scale bar-500nm). High magnification micrograph focuses on MVBs harboring smaller vesicles of different sizes and shapes (TEM was performed on three independent tumor tissues).

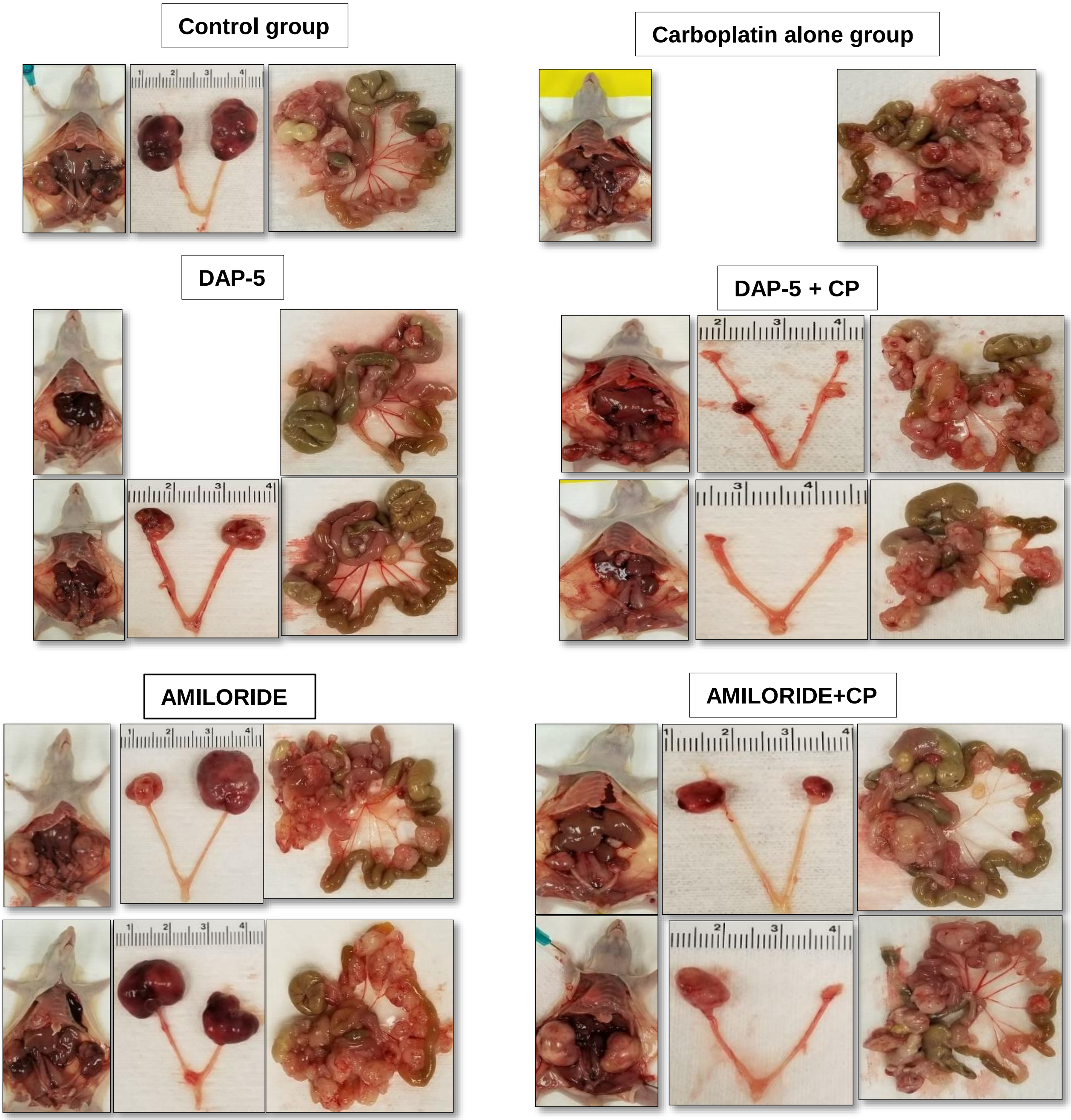

Supplementary Figure 11: Orthotopic ovarian tumors were developed in nude mice by injecting the TR127 cells into the ovarian bursa to investigate the potential of carboplatin alone 2 mg/Kg.b.wt/week (n=5 mice/group±SD) compared with control mice (no treatment) for four weeks starting 7 days after the implantation of tumor cells. ISG15 inhibitor (DAP5-100ppm in animal feed daily alone and in combination with carboplatin therapy (2mg/Kg.b.wt/week (n=5 mice/group±SD) for four weeks starting 7 days after the implantation of tumor cells. Potential of exosome inhibitor (amiloride 2mg/Kg b.wt/weekly twice) alone and in combination with carboplatin therapy (2mg/Kg.b.wt/ week, n=5 mice/group±SD) for four weeks starting 7 days after the implantation of tumor cells.

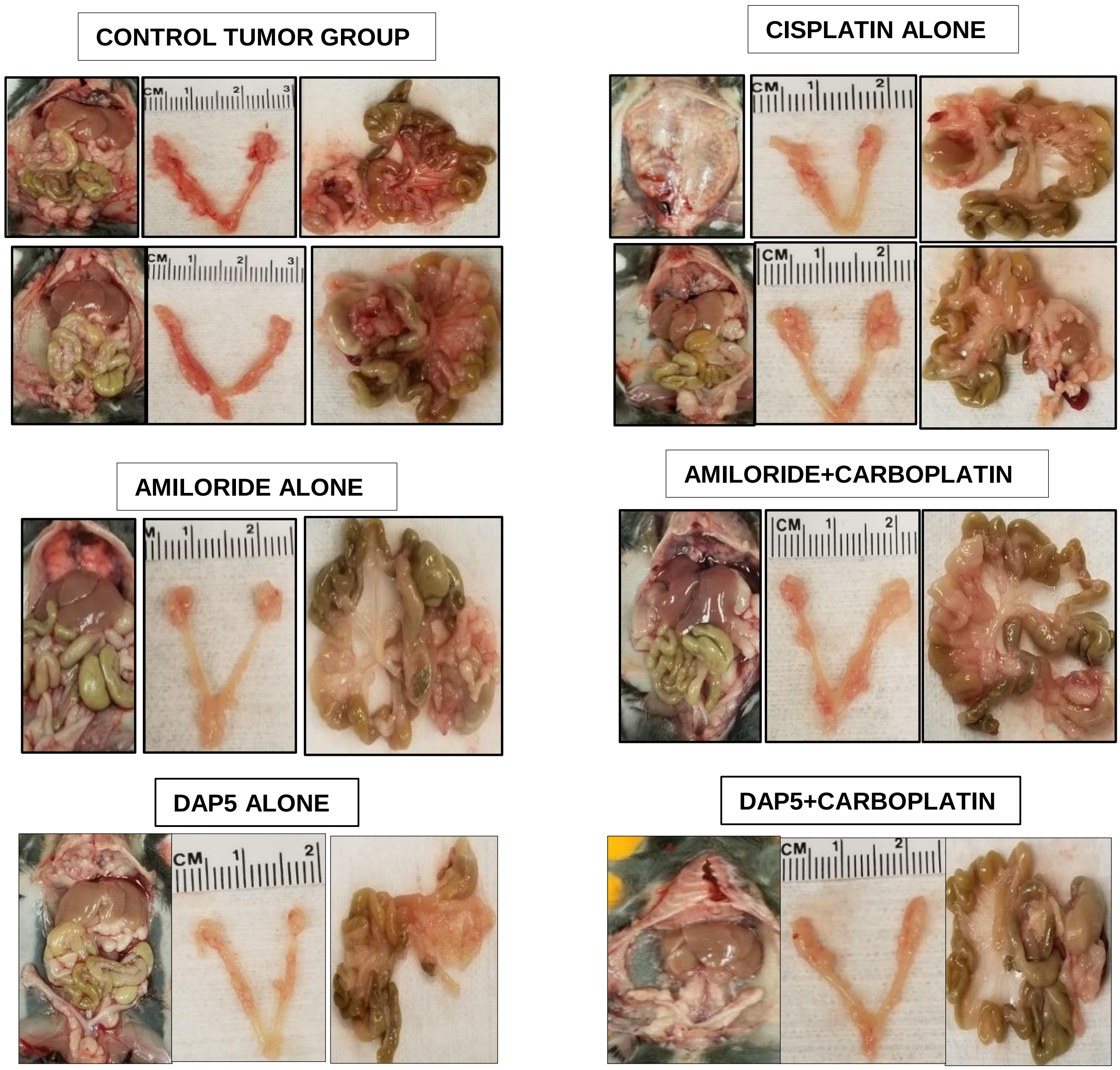

Sup. Fig. 12: Orthotopic ovarian tumors were developed in immunocompetent mice by injecting the mouse ovarian cancer cells ID8 into the ovarian bursa to investigate the potential of carboplatin therapy(2mg/Kg.b.w/ week, n=4mice/group±SD) alone for four weeks starting 7 days after the implantation of tumor cells and compared with the control tumor group (untreated). Exosome inhibitor (amiloride -2mg/Kg.b.wt/weeky twice) alone and in combination with carboplatin therapy (2mg/Kg.b.w/ week) (n=3mice/group±SD) for four weeks starting 7 days after the implantation of tumor cells. Small molecule inhibitor–DAP5 to inhibit ISG15 (dose-100ppm in animal feed daily) alone and in combination with carboplatin therapy(2mg/Kg.b.w/ week, n=3mice/group±SD) for four weeks starting 7 days after the implantation of tumor cells.

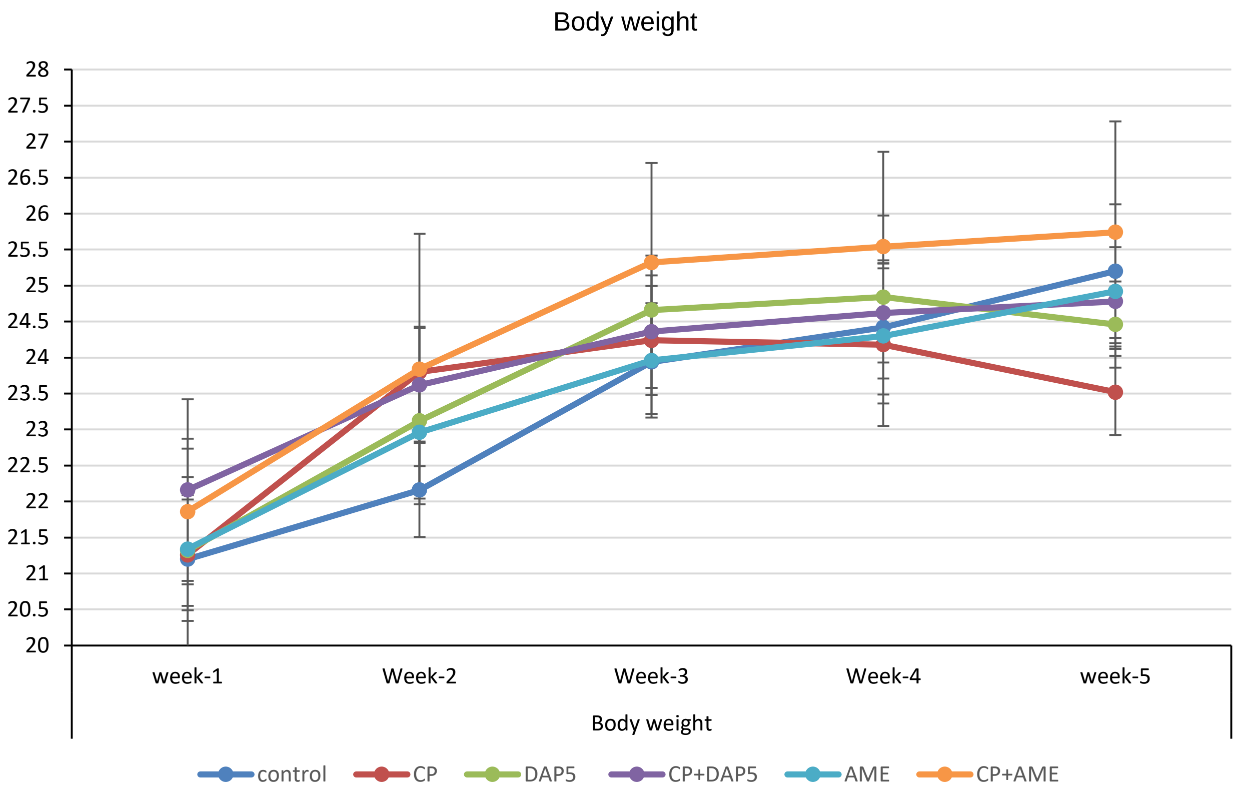

Supplementary Figure 13: Effect of DAP5 or exosome inhibitor (Amiloride) in combination with carboplatin (weekly i.p. injection of 2mg/kg) on body weight. Data obtained 4 weeks after treatment. DAP5 significantly enhanced the cytotoxic/ cytostatic effect of carboplatin in ID8 tumor cells without significantly affecting the body weight.

(a)

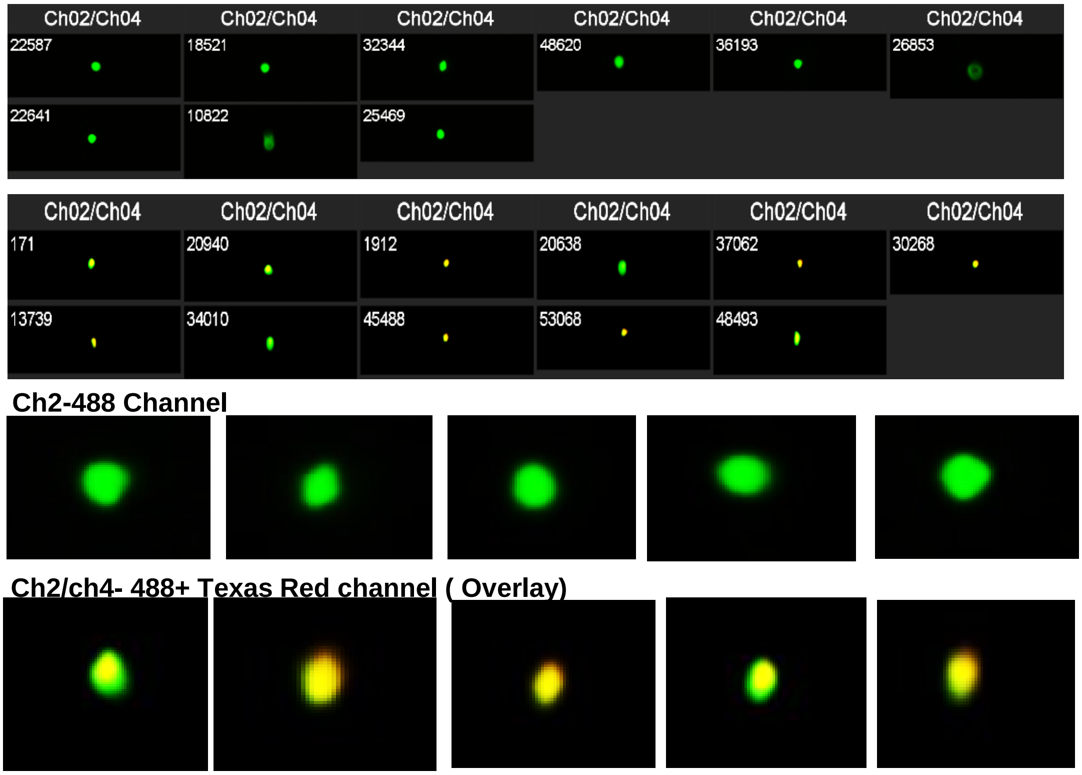

(b)

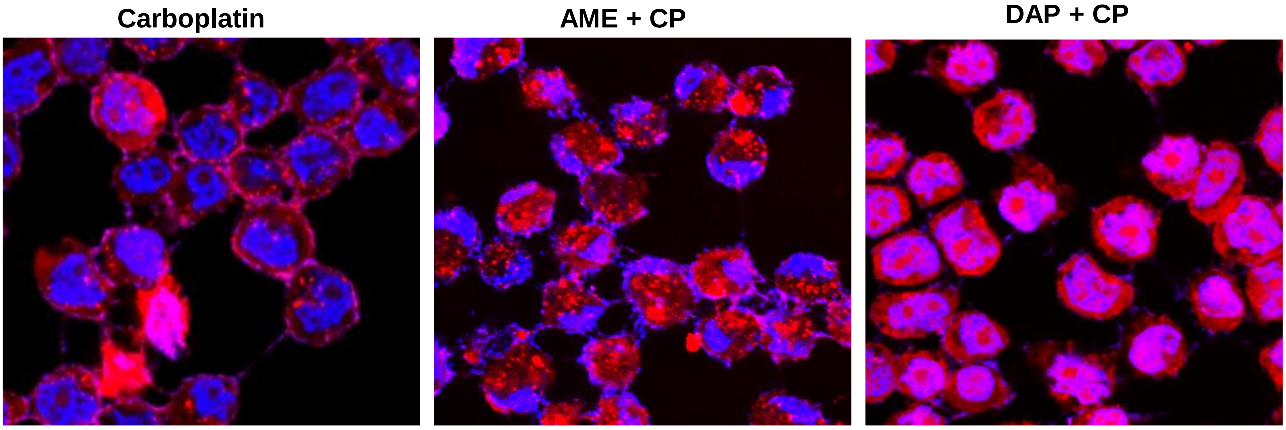

Supplementary Figure 14: a) Image stream analysis of vesicles captured in the flow showing single FITC positive vesicle on 488 Channel(Ch2) overlaid for Texas red positivity in the 564 channel (Ch4) confirming the efflux of Tr-CP labelled cisplatin via the vesicles in POCC cells. **b)** When treated with Texas-red-labeled CP, the Tr127 cells showed increased accumulation of cisplatin (5uM) within the cells when treated in combination with (DAP5-5uM or AME-10uM) for 12h. Increased accumulation of CP is clearly visible in the nucleus and cytoplasm accumulation of the POCCs counterstained with DAPI /blue for nucleus.

CA125

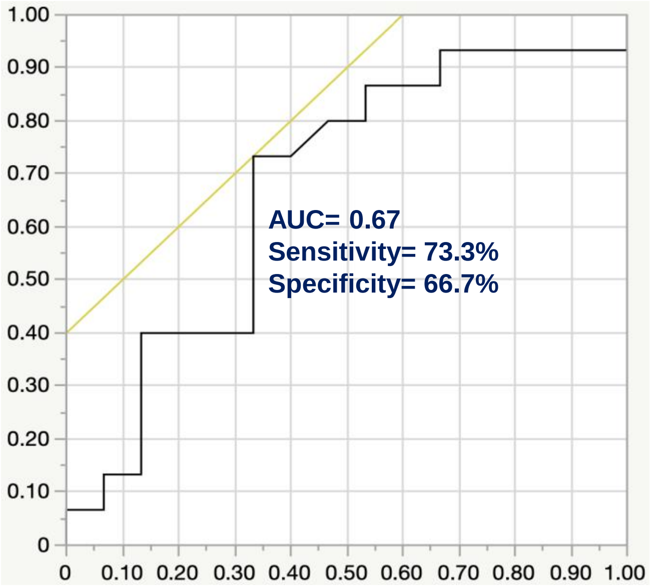

ISG15

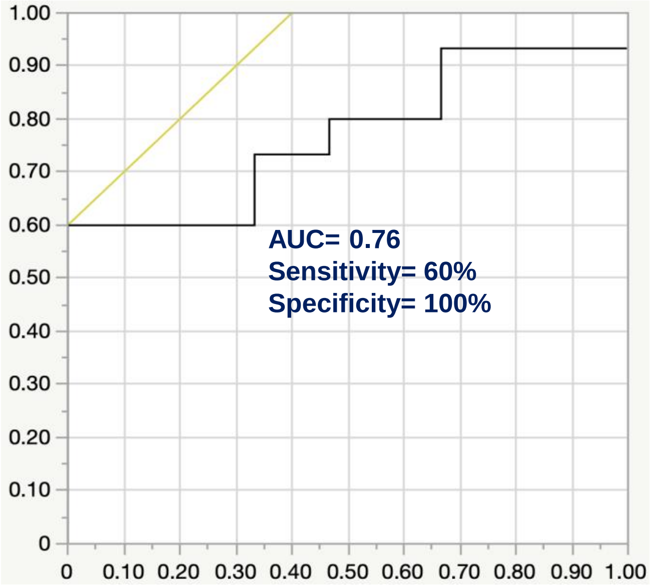

Supplementary Figure 15: The ROC curve and AUC for CA125 and ISG15 in whole serum of control and advanced HGSOC patients.
